# Adipocyte-like signature in ovarian cancer minimal residual disease identifies metabolic vulnerabilities of tumor-initiating cells

**DOI:** 10.1172/jci.insight.147929

**Published:** 2021-06-08

**Authors:** Mara Artibani, Kenta Masuda, Zhiyuan Hu, Pascal C. Rauher, Garry Mallett, Nina Wietek, Matteo Morotti, Kay Chong, Mohammad KaramiNejadRanjbar, Christos E. Zois, Sunanda Dhar, Salma El-Sahhar, Leticia Campo, Sarah P. Blagden, Stephen Damato, Pubudu N. Pathiraja, Shibani Nicum, Fergus Gleeson, Alexandros Laios, Abdulkhaliq Alsaadi, Laura Santana Gonzalez, Takeshi Motohara, Ashwag Albukhari, Zhen Lu, Robert C. Bast, Adrian L. Harris, Christer S. Ejsing, Robin W. Klemm, Christopher Yau, Tatjana Sauka-Spengler, Ahmed Ashour Ahmed

**Affiliations:** 1 Ovarian Cancer Cell Laboratory, Medical Research Council (MRC) Weatherall Institute of Molecular Medicine, and; 2 Nuffield Department of Women’s & Reproductive Health, University of Oxford, Oxford, United Kingdom.; 3 Gene Regulatory Networks in Development and Disease Laboratory, MRC Weatherall Institute of Molecular Medicine, Radcliffe Department of Medicine, Medical Sciences Division, University of Oxford, Oxford, United Kingdom.; 4 Department of Molecular Life Sciences, University of Zurich, Zurich, Switzerland.; 5 Department of Gynaecological Oncology, Churchill Hospital, Oxford University Hospitals, Oxford, United Kingdom.; 6 Department of Oncology, Medical Sciences Division, University of Oxford, Oxford, United Kingdom.; 7 Department of Histopathology, Oxford University Hospitals, Oxford, United Kingdom.; 8 Department of Radiology, Churchill Hospital, Oxford University Hospitals, Oxford, United Kingdom.; 9 Department of Obstetrics and Gynecology, Faculty of Life Sciences, Kumamoto University, Chuo-ku, Kumamoto, Japan.; 10 Department of Biochemistry, Faculty of Science, King Abdulaziz University, Jeddah, Saudi Arabia.; 11 Department of Experimental Therapeutics, University of Texas MD Anderson Cancer Center, Houston, Texas, USA.; 12 Department of Biochemistry and Molecular Biology, VILLUM Center for Bioanalytical Sciences, University of Southern Denmark, Odense, Denmark.; 13 Cell Biology and Biophysics Unit, European Molecular Biology Laboratory, Heidelberg, Germany.; 14 Department of Physiology, Anatomy and Genetics, Medical Sciences Division, University of Oxford, Oxford, United Kingdom.; 15 Division of Informatics, Imaging and Data Sciences, School of Health Sciences, Faculty of Biology Medicine and Health, the University of Manchester, Manchester, United Kingdom.; 16 Oxford National Institute for Health Research (NIHR) Biomedical Research Centre, Oxford, United Kingdom.

**Keywords:** Oncology, Fatty acid oxidation, Obstetrics/gynecology

## Abstract

Similar to tumor-initiating cells (TICs), minimal residual disease (MRD) is capable of reinitiating tumors and causing recurrence. However, the molecular characteristics of solid tumor MRD cells and drivers of their survival have remained elusive. Here we performed dense multiregion transcriptomics analysis of paired biopsies from 17 ovarian cancer patients before and after chemotherapy. We reveal that while MRD cells share important molecular signatures with TICs, they are also characterized by an adipocyte-like gene expression signature and a portion of them had undergone epithelial-mesenchymal transition (EMT). In a cell culture MRD model, MRD-mimic cells showed the same phenotype and were dependent on fatty acid oxidation (FAO) for survival and resistance to cytotoxic agents. These findings identify EMT and FAO as attractive targets to eradicate MRD in ovarian cancer and make a compelling case for the further testing of FAO inhibitors in treating MRD.

## Graphical abstract

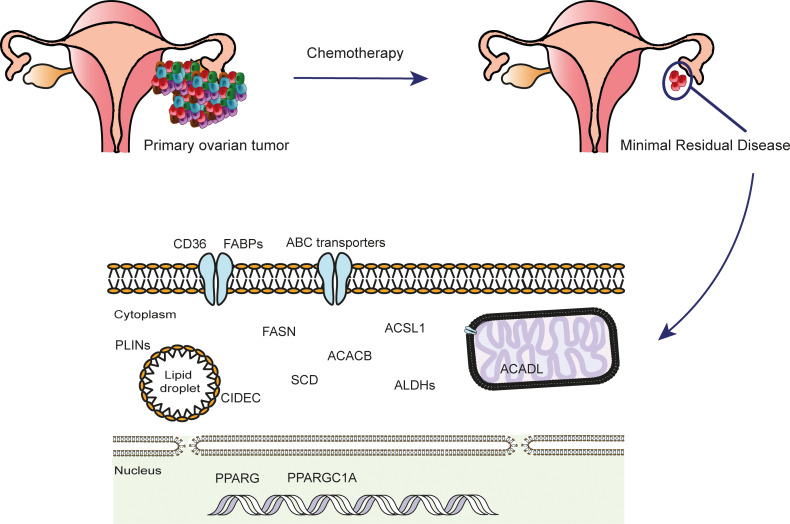

## Introduction

The term “minimal residual disease” (MRD) was originally coined in relation to hematological malignancies to define the leukemic cells that remain after treatment (1). More generally, tumor MRD describes cancer cells that remain following complete clinical and radiological response to therapeutic interventions (2). Such cancer cells share phenotypic and genomic characteristics with the bulk tumor that existed prior to the intervention. In hematological malignancies such as chronic myeloid leukemia and acute lymphoblastic leukemia, personalized treatment of MRD demonstrated the feasibility of achieving long-term responses and cures, presumably by eliminating residual cancer cells (3–5). Importantly, these examples have shown that rationalized switching of treatment to circumvent the development of resistance in MRD can still help achieve long-term benefits in patients.

However, the concept of treating MRD in solid tumors remains largely unexplored because of limited understanding of the drivers of MRD survival. Current knowledge is largely obtained from preclinical models rather than directly from patients and suggests that MRD survival is related to key characteristics that define tumor-initiating cells, such as overexpression of ATP-binding cassette (ABC) transporters and overactivity of aldehyde dehydrogenases (ALDHs) (6). However, the clinical relevance of these observations and the presence of any additional survival mechanisms for MRD in patients has remained unknown because of the difficulty in isolating and characterizing MRD cells.

It is important to make a distinction between measuring the MRD load and the molecular characterization of MRD cells. The former is largely a diagnostic process for predicting the probability of recurrence while the latter aims to understand driving survival pathways and potential tumor vulnerabilities for therapeutic intervention (2). Recent advances in isolating and quantifying circulating tumor DNA and circulating tumor cells, among other technologies, have made it possible to predict the load of MRD and the probability of recurrence with high precision (7, 8). These methods could also be extended to detect the presence or evolution of known mechanisms of resistance to therapies such as the development of resistance mutations in the active site of a kinase that is being targeted therapeutically (9). However, such approaches would only be helpful for predicting response to a limited number of therapeutics (2). Conversely, an unbiased molecular characterization of MRD would be ideal for the discovery of novel therapeutic strategies to treat MRD. In many hematological malignancies, it is possible to sample MRD by obtaining bone marrow biopsies. In contrast, the selection of the biopsy sites to harvest MRD from solid tumors is much more challenging since it is difficult to predict their site of residence.

To overcome the aforementioned limitations, we designed a prospective observational study in patients with advanced high-grade serous ovarian cancer (HGSOC) allowing us to sample and characterize MRD. Given HGSOC’s relatively short latency before recurrence and its tendency not to spread outside of the abdominal cavity, sampling MRD from the peritoneal cavity provides an opportunity to characterize clinically relevant MRD lesions. To define MRD, we applied strict criteria of complete responses that are based on clinical and radiological evidence, direct visualization of the peritoneal cavity, and histopathological evidence of significant response. We used laser capture microdissection (LCM) to enable further in-depth characterization of MRD sites. This work enabled us to identify a highly selected and pure population of tumor cells that faithfully represent MRD directly in patients. Our results provide potential opportunities for therapeutic intervention to treat MRD in patients with HGSOCs.

## Results

### The Oxford Ovarian Cancer Predict Chemotherapy Response study patient cohort.

The study of MRD in ovarian cancer or other solid tumors in patients has been difficult because of the inability to identify and sample microscopic deposits intraoperatively or by using traditional imaging modalities. To overcome this limitation, we designed a prospective observational study, Oxford Ovarian Cancer Predict Chemotherapy Response (OXO-PCR), to enable intraoperative identification and sampling of MRD in ovarian cancer patients. We used the combination of video-laparoscopy and surgical clip application prior to the start of chemotherapy to mark tumor areas from which samples were obtained. Following chemotherapy, we then recollected samples from either the tissue surrounding the surgical clips or from spatially related areas as guided by the prechemotherapy video recording irrespective of whether a tumor was visible (Supplemental Video 1; supplemental material available online with this article; https://doi.org/10.1172/jci.insight.147929DS1). These samples were then sectioned and examined microscopically to identify MRD and, when found, laser capture microdissected for further analysis.

The OXO-PCR study was conducted prospectively in Oxford, United Kingdom, from 2012 to 2017. We recruited 17 patients with at least stage IIIC HGSOC. Overall, the cohort had a median age of 71 years at the time of diagnosis, a median time to relapse of 11 months from completion of first-line treatment, and an average survival of 22 months. All patients received at least 3 cycles of the standard carboplatin-paclitaxel combination treatment, except 2 patients who received carboplatin alone due to hypersensitivity to paclitaxel (Supplemental Table 1). For each patient, paired samples were obtained before and after treatment from multiple metastatic tumor sites, first at the time of diagnostic laparoscopy (prior to chemotherapy), and second, during interval debulking surgery (IDS) (following at least 3 cycles of neo-adjuvant chemotherapy, NACT).

**Figure 1:**
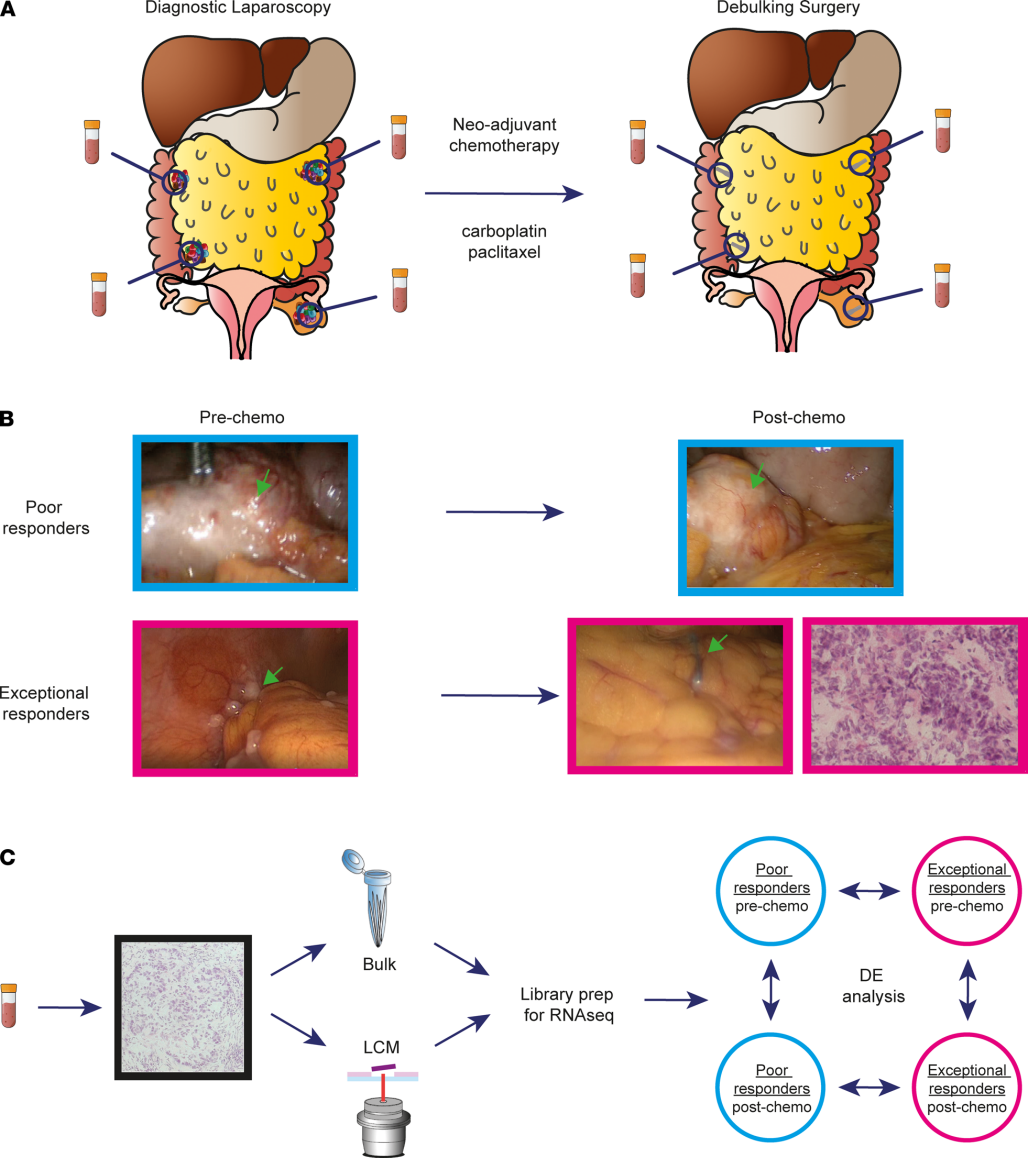
Intraoperative identification and sampling of MRD in ovarian cancer patients. (**A**) Diagram shows the sampling technique used in the OXO-PCR study. All 17 patients had paired biopsies collected at the time of diagnostic laparoscopy (pre-chemo) and during the IDS that followed at least 3 cycles of NACT (post-chemo). (**B**) Representative images showing the tumor burden in poor and exceptional responders before and after treatment. The MRD cancer islets are not visible during the IDS and can only be detected with a hematoxylin-and-eosin staining of the biopsy. (**C**) Diagram shows the RNA-Seq pipeline. Each biopsy was cryosectioned, stained, and assessed by a gynecological oncology pathologist to confirm presence of cancer cells; RNA-Seq libraries were prepared from both bulk and laser capture microdissected material, followed by differential expression analysis across time points and response groups.

Applying the above sampling technique in individual patients ensured paired sampling from the same sites at 2 time points, allowing for intrapatient as well as interpatient heterogeneity to be studied over time (Figure 1A).

Overall chemotherapy response was evaluated using CT scans and cancer antigen 125 blood levels, while the site-specific response was assessed through laparoscopies that were conducted before and after chemotherapy (10). Based on these criteria, patients were divided into 3 groups: “exceptional responders” if all the metastatic sites showed a complete clinical response following primary treatment and only microscopic tumor foci were found in the biopsies collected during IDS (MRD), “poor responders” if all the sites showed extensive macroscopic disease after NACT, and “mixed responders” if patients had both MRD sites and poor response sites (Figure 1B and Supplemental Table 1A).

In order to compare the gene expression profiles across response groups, RNA-Seq was performed on cancer islets isolated using LCM from all samples collected at both time points. Due to the abundance of tumor cells before treatment and in the “postchemo” samples in sites where there was evidence of poor response, scrolls of their samples were also collected to be used for bulk RNA-Seq analyses (Figure 1C). Both LCM and bulk RNA-Seq pipelines included multiple quality control steps to avoid the contamination from surrounding noncancer tissue (Supplemental Figure 1, A and B). After quality control filtering (which removed 37 out of the total 156 libraries), differential expression analyses were carried out across time points and response groups as well as on each patient individually (Figure 1C).

### Pseudotime analysis reveals limited intrapatient heterogeneity.

**Figure 2:**
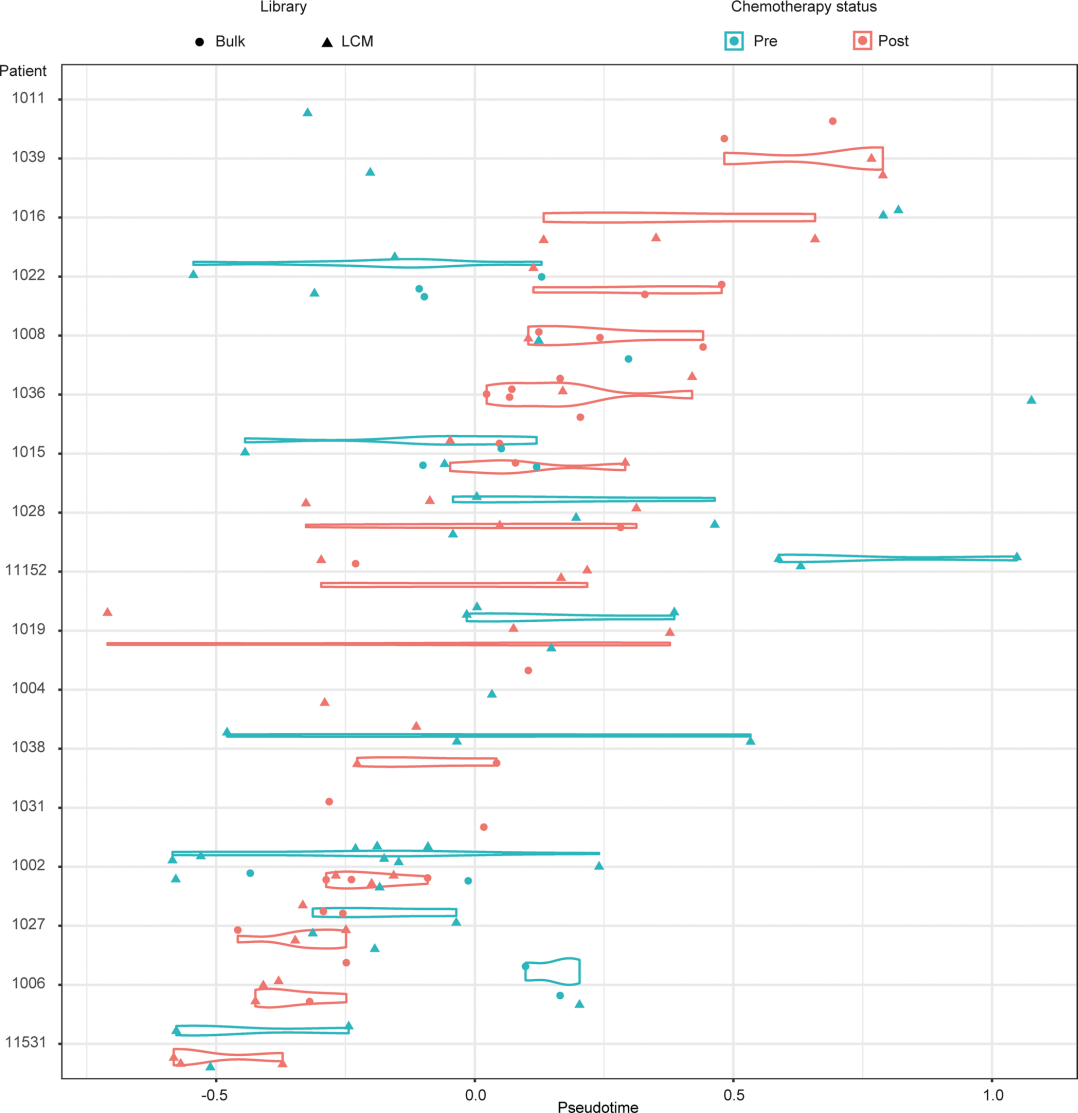
Pseudotime analysis reveals limited intrapatient heterogeneity. Pseudotime analysis shows that samples from the same patient cluster together on the pseudotime gradient. Patients 1016, 1036, and 11152 are exceptional responders; patients 1015, 1038, and 1006 are poor responders.

We first sought to evaluate how representative the sample set is to the known molecular profile of HGSOC. To do this, we used unsupervised pseudotime analysis (11) of the prechemotherapy sample set and compared this to data obtained from the The Cancer Genome Atlas (TCGA) HGSOC data set (12), which comprised, predominantly, prechemotherapy samples. This comparison revealed that our set clustered around the center of the pseudotime gradient of the TCGA cohort (Supplemental Figure 2A), indicating that the data set is highly representative of HGSOCs. Next, we examined the pseudotime data of the entire data set (prechemo and postchemo) and found that samples from the same patient clustered together on the pseudotime gradient (Figure 2) despite the analysis being conducted in an unsupervised manner without taking into account the patient identity or timing of sampling. This result was also consistent with the t-distributed stochastic neighbor embedding of the entire data set following batch correction (Supplemental Figure 2B), strongly suggesting that, despite the existence of intrapatient heterogeneity, the gene expression diversity observed in the OXO-PCR cohort is clearly dominated by interpatient heterogeneity.

We next identified pseudotime-dependent genes by fitting a linear model and highlighting genes that are differentially expressed along the pseudotime gradient independent from chemotherapy effect. Pathway analysis revealed an enrichment of genes that are involved in interferon signaling, L1CAM, and MAPK pathways (Supplemental Figure 2C). Importantly, after accounting for the pseudotime effect, and, as expected, analysis of the chemotherapy effect revealed a significant downregulation in FOXM1 expression, a master regulator of the expression of genes involved in mitosis (12). There was also evidence of downregulation of the corresponding mitotic signature that is known to be highly expressed in HGSOC (12), with downregulation of known mitosis genes such as AURKB, NCAPH, NCAPG, Cyclin B2, and several kinesins (Supplemental Table 2). Overall, these data show the robustness of the approach chosen for the study and highlight that transcriptional heterogeneity is predominantly observed between patients rather than within individual patients.

### Transcriptomic signatures related to tumor-initiating cells and lipid metabolism characterize HGSOC MRD cells.

**Figure 3:**
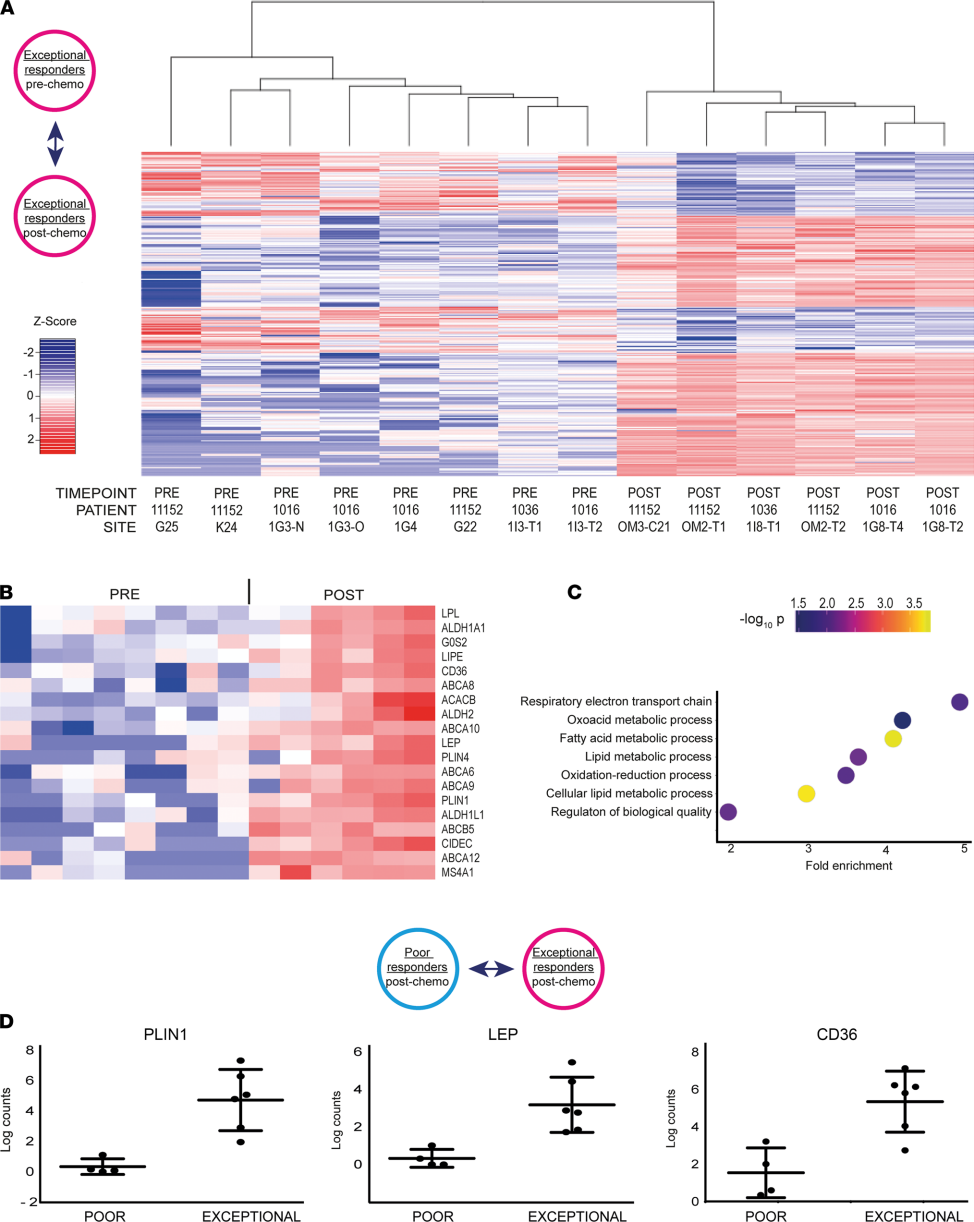
LCM-guided RNA-Seq of HGSOC MRD cells identifies specific adipocyte-like and tumor-initiating cell signatures. (**A**) Heatmap shows the 356 differentially expressed genes obtained comparing the transcriptomes of exceptional responders before and after treatment. (**B**) Heatmap shows selected genes from the adipocyte-like and TIC signatures upregulated in MRD. The order of the samples is the same used in **A**. *LEP*, leptin; *PLIN1*, perilipin 1. (**C**) Dot plot shows the main biological processes enriched in the postchemotherapy samples of the exceptional responder patient 1016. (**D**) Graphs show expression levels of genes from the adipocyte-like signature in poor and exceptional responders after treatment.

We next sought to evaluate MRD in exceptional responders (patients 11152, 1016, and 1036), who all had no visible residual disease at the postchemotherapy laparoscopy. We compared the gene expression profiles of LCM samples obtained before and after treatment from these tumors (Figure 3A). This analysis identified 356 differentially expressed genes (Figure 3A and Supplemental Table 3).

The postchemotherapy tumor cells showed significantly higher expression of ABC transporters (*ABCA12*, *ABCB5*, *ABCA9*, *ABCA6*, *ABCA10*, *ABCA8*; log fold change > 3.3; *P* < 1.68 × 10^4^) as well as other known markers of tumor-initiating cells (TICs; *ALDH1L1*, *ALDH1A1*, *ALDH2*, *MS4A1/CD20*; log fold change > 1.8; *P* < 9.4 × 10^4^) (Figure 3B) (13, 14).

These results suggest that MRD has characteristics that are consistent with previously identified features of TICs from preclinical models. Surprisingly, we also found a significant increase in the expression of genes that are involved in lipid metabolism, such as *PLIN1*, *PLIN4*, *CD36*, *ACACB*, *G0S2*, *LIPE*, *LPL*, *GPAM*, and *SCD* (Figure 3B) (log fold change > 3.7, *P* < 2.1 × 10^4^). Notably, the MRD cells were also characterized by the upregulation of 60 small nucleolar RNAs (snoRNAs; Supplemental Table 3), noncoding RNAs whose traditional role is to guide the posttranscriptional modification of ribosomal and small nuclear RNAs. More recently, however, snoRNAs have been shown to also play important roles in tumorigenesis and in the regulation of lipotoxic and oxidative stress responses (15).

Importantly, the expression of HGSOC marker genes such as *PAX8*, *MUC16*, and *WT1* or the epithelial marker *EPCAM* was maintained after chemotherapy, indicating that the MRD LCM cells kept their HGSOC identity (Supplemental Figure 3A).

Moreover, to rule out the possibility of cross-contamination of MRD with adipocytes, we attempted to perform LCM and RNA-Seq on large areas of adipose tissue adjacent to the MRD lesions and compare the RNA expression results. However, this did not yield sufficient RNA for downstream analysis. We conclude that the possibility of contamination of MRD with a small number of adipocytes is highly unlikely to have biased the differential expression analysis.

We next examined the differentially expressed genes per individual patient from the exceptional responders. We noted that for patient 1016, who had the most notable microscopic response, the main biological processes that were enriched in postchemotherapy samples were related to fatty acid metabolism (*P* < 0.001, FDR < 0.001) (Figure 3C). The differentially overexpressed genes are known to be involved in the uptake (*CD36*, *FABP4*, *FABP5*), storage (*PLIN1*, *PLIN4*, *PLIN5*), synthesis (*FASN*, *FADS3*, *ACACB*, *SCD*), and oxidation (*ACADL*, *ACSL1*) of fatty acids (Supplemental Figure 3B).

These genes, or genes belonging to the same pathways, were also significantly overexpressed in postchemotherapy samples of patients 1036 (*FABP2*, *FABP4*, *PLIN1*, *PLIN4*, *SCD*, *ACADL*, *ACSL6*) (Supplemental Figure 3C) and 11152 (*ACADSB*; relaxing the FDR from 0.05 to 0.1 also *CD36*, *PPARGC1A*, *PLIN1*) (Supplemental Figure 3D). These findings were further confirmed by quantitative real-time PCR using a subset of upregulated genes (Supplemental Figure 3E). In addition to the lipid metabolism markers, the MRD islets of patient 1016 also showed modulation of genes, such as *AMOT*, belonging to a dormancy signature previously identified in breast cancer (16) (Supplemental Figure 3B), suggesting the presence of a subpopulation of more quiescent cells within the captured MRD sample.

To determine whether the features described above are specific to MRD or shared among all chemotherapy-resistant cells, we compared the LCM data from the exceptional and the poor responders after treatment. Even though both cell populations survived NACT, significant transcriptional differences were observed with 867 genes found to be differentially expressed between the 2 groups. Notably, the MRD samples showed upregulation of genes related to lipid metabolism and those previously known to be associated with TICs (Figure 3D and Supplemental Figure 3F) (log fold change > 2.7, *P* < 0.001) as well as snoRNAs (Supplemental Table 4). These results further support the notion that fatty acid metabolism is specifically upregulated in MRD, highlighting a potentially previously unrecognized feature of these cancer cells in patients with HGSOC.

### The transcriptome of MRD cells resembles differentiated adipocytes.

**Figure 4:**
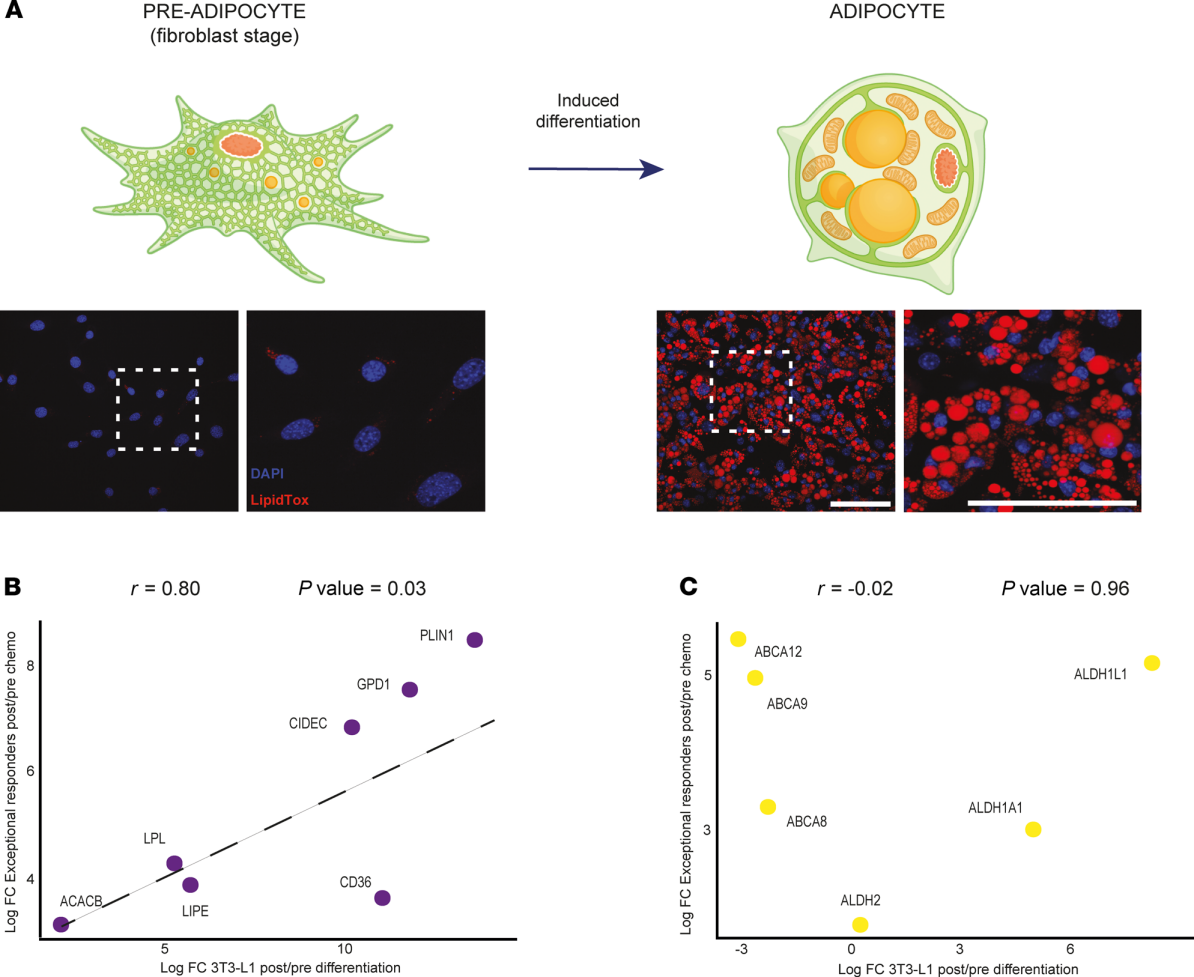
The transcriptome of MRD cells resembles differentiated adipocytes. (**A**) Diagram in the upper panel represents the differentiation of 3T3-L1 cells into adipocytes. In the lower panel, fluorescence images with LipidTox staining show lipid droplets’ accumulation upon differentiation. Scale bars: 100 μm. (**B**) Scatterplot shows a positive correlation for lipid metabolism genes between the log_2_ fold change (log_2_FC) observed in the exceptional responders (post/pre chemo) and the log_2_FC in the 3T3-L1 differentiation experiment (post/pre expression ratios). (**C**) Scatterplot shows absence of correlation for ABC transporters and TIC genes between the log_2_FC observed in the exceptional responders (post/pre chemo) and the log_2_FC in the 3T3-L1 differentiation experiment (post/pre expression ratios).

The observation that the identified MRD-upregulated genes are involved in both anabolic and catabolic lipid metabolic processes implied that the purpose of such upregulation was not to simply increase ATP uptake following chemotherapy. Instead, these observations pointed to a more complex transcriptional program of MRD cancer cells that may contribute to the acquisition of chemotherapy resistance. These transcriptional changes were reminiscent of those observed in adipocytes, where both synthesis and turnover of storage lipids such as triacylglycerols are highly active. To test the hypothesis that MRD cancer cells selected by chemotherapy are adipocyte like, we compared the transcriptional changes to those that occur during differentiation of adipocytes from fibroblast-like precursors. To this end, we differentiated 3T3-L1 cells into adipocytes as previously described (17). To monitor transcriptional changes, we performed RNA-Seq at day –2 (preadipocyte/fibroblast stage), day 0 (start of the differentiation protocol), and day 6 (adipocyte stage) (Figure 4A). Strikingly, we found that the expression of key adipocyte markers such as *CD36*, *PLIN1*, *CIDEC*, *LIPE*, *LPL*, and *ACACB*, all strongly upregulated upon adipocyte differentiation (Supplemental Figure 4), correlated significantly with the expression changes observed in exceptional responders (Pearson’s correlation coefficient of 0.8, *P* value of 0.03) (Figure 4B). In contrast, overexpression of genes that were known to be upregulated in TICs such as ABC transporters was observed only in MRD (Figure 4C) and not during adipocyte differentiation. These results strongly suggest that MRD cancer cells, while retaining features of TICs, reflect a transcriptional state that resembles adipocytes.

Altogether, these findings represent the first in vivo characterization to our knowledge of MRD cells isolated from HGSOC patients and identify specific markers that are unique to this population of chemotherapy-resistant cells.

### MRD cells show mesenchymal characteristics.

We observed that the resistant cancer cells that were laser captured from exceptional responders prior to RNA-Seq showed an elongated and spindle-like shape in contrast to the more rounded appearance of cancer cells isolated from poor responders. The appearance of MRD cancer cells was consistent with that of mesenchymal cells, suggesting that they may represent epithelial-to-mesenchymal transition (EMT). This was a reasonable assumption given that chemotherapy resistance (18, 19) and the acquisition of stem cell properties (20, 21) have been clearly associated with cancer-related EMT.

**Figure 5:**
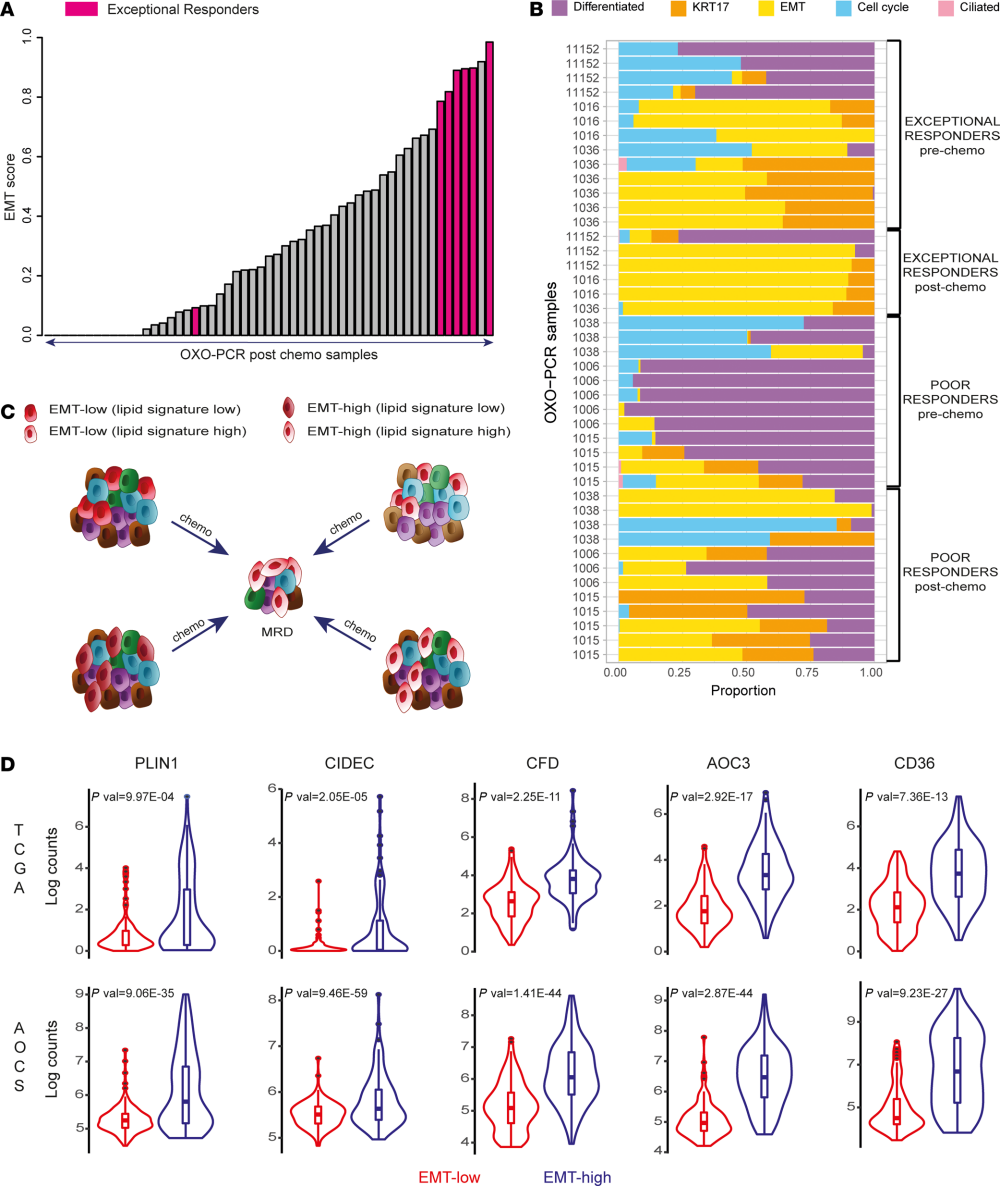
HGSOC MRD cells display EMT features. (**A**) Bar plot shows the EMT score of all the postchemo OXO-PCR samples calculated using our deconvolution-based classifier (Methods). (**B**) Stacked bar plot visualizes the deconvolution result of 44 bulk and LCM tumor samples collected from 6 patients (3 poor responders and 3 exceptional responders). Colors of the bars denote the 5 cell states as shown in the legend. (**C**) The diagram presents alternative models to explain the adipocyte-like state observed in MRD. The lipid metabolism signature could be selected upon treatment (top left, bottom right), with either the coexistence of lipid-high and EMT-high phenotypes in the same cells (bottom right) or not (top left) before chemotherapy. Alternatively, the adipocyte-like state may be induced by chemotherapy (top right, bottom left), and the EMT features may be already present before treatment (top right) or not (bottom left) before chemotherapy. The different colors are used to represent tumor heterogeneity and possible clonal populations. (**D**) Violin plots show the expression levels of lipid metabolism genes in the EMT-high samples compared with the EMT-low ones across the TCGA and AOCS data sets (*P* values were computed by limma voom).

To test this assumption, we next quantified the EMT cell state in the OXO-PCR samples using our recently described deconvolution-based classifier (22, 23). This analysis revealed that MRD cells are particularly enriched in genes belonging to the EMT signature. Specifically, among all the postchemotherapy samples, those isolated from exceptional responders showed the highest EMT score (Figure 5A). These MRD samples were characterized by a very high proportion of the EMT-high cell state (EMT fraction > 0.85) compared with other cell states, regardless of whether a high EMT level was already widely observed before treatment (as in patient 1016) or not (as in patients 11152 and 1036) (Figure 5B). In contrast, the samples from poor responders were more heterogeneous after chemotherapy, showing the coexistence of multiple cell states (e.g., EMT-high, differentiated, Krt17, cell cycle) (Figure 5B and Supplemental Figure 5).

Given the EMT enrichment in MRD, we reasoned that the adipocyte-like gene signature observed in MRD may be a defining feature of the EMT-high cancer cell state, selected in the chemotherapy-resistant, EMT-high MRD cells (Figure 5C). The alternative explanation is that the adipocyte-like state, the EMT-like state, or both are induced by chemotherapy (Figure 5C). To test these alternatives, we compared the adipocyte-like gene signature between EMT-high and EMT-low prechemotherapy tumors using publicly available data sets of prechemotherapy HGSOC, TCGA (12), and the Australian Ovarian Cancer Study (AOCS) (24). We divided the samples according to our previously described EMT score (22, 23) into EMT-high and EMT-low.

Our analysis indicated that many genes from the adipocyte-like gene signature showed significantly higher expression in the EMT-high group (Figure 5D). This suggests that EMT-high cancer cells are enriched in genes related to lipid metabolism and that this cell state becomes selected for after chemotherapy treatment. However, our results cannot completely rule out the alternative explanations that the adipocyte-like gene signature or the EMT-high signature are, at least in part, induced by chemotherapy.

Collectively, these data highlight the mesenchymal characteristics of MRD that encompass an elevated adipocyte-like signature. We speculate that active lipid metabolism might confer a survival advantage for chemotherapy-resistant MRD.

### MRD-mimic cells in vitro are sensitive to inhibitors targeting fatty acid oxidation.

To functionally characterize the MRD population, we developed an in vitro model of MRD that successfully recapitulates the key gene expression features observed in vivo.

**Figure 6:**
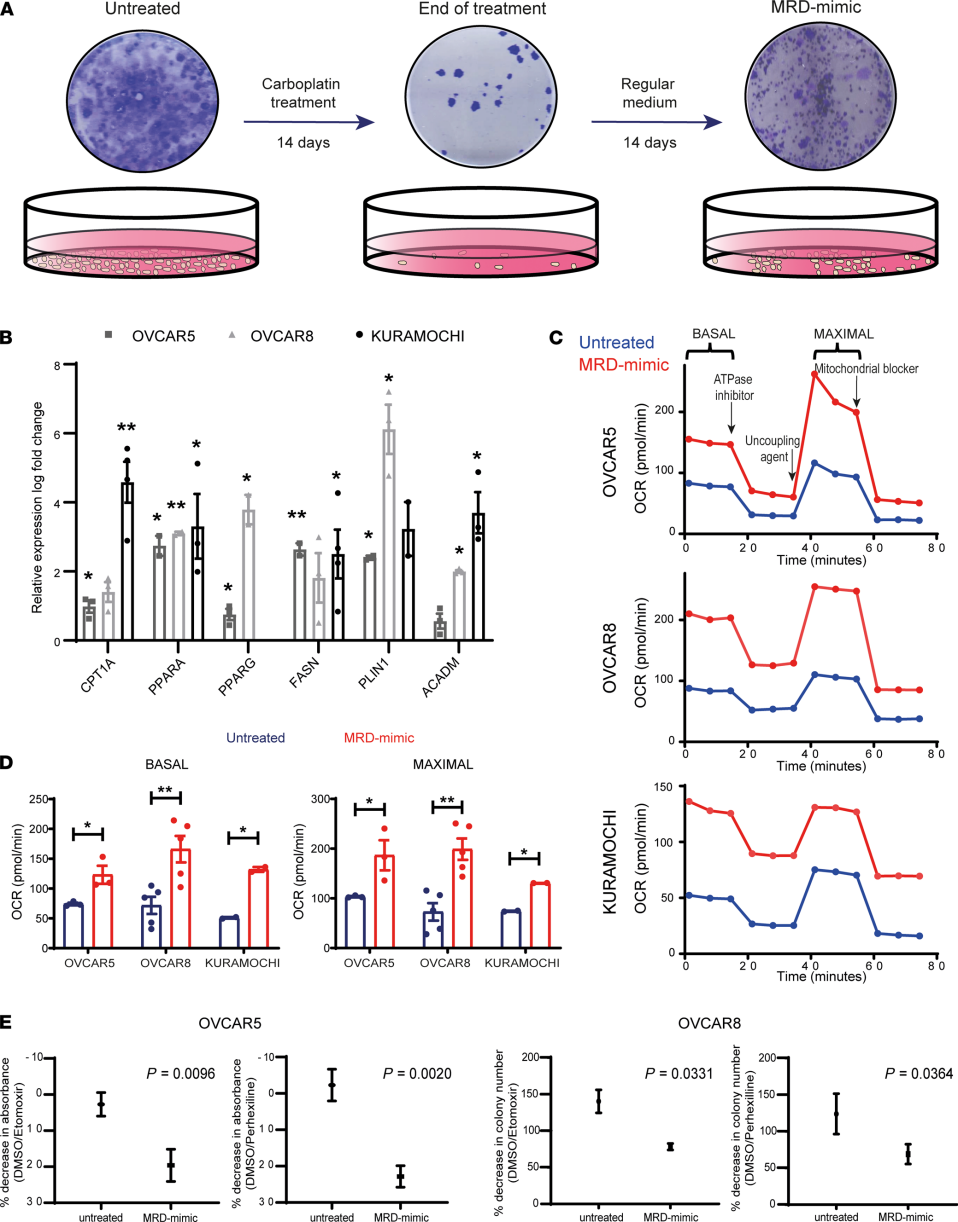
An in vitro model reveals that fatty acid oxidation is required for MRD survival. (**A**) Diagram showing the MRD in vitro model. Cells were treated for 2 weeks with carboplatin concentrations that achieved more than 90% cell killing (end-of-treatment time point), after which the surviving cells were allowed to recover in regular medium for an additional 2 weeks (MRD-mimic time point). (**B**) Quantitative real-time PCR of genes from the lipid signature in MRD-mimic cells. The graph represents log fold change of mean expression relative to untreated cells; error bars represent the standard deviation from *n* = 3 biological replicates. A 2-tailed *t* test was used to calculate the *P* values (*P < 0.05, **P < 0.01). (KURAMOCHI cells do not express *PPARG*.) (**C** and **D**) Representative pattern of OCR as a function of time (min) normalized to DNA content in untreated and MRD-mimic cells (**D**). Bar plots show means ± SEM basal (left) and maximal (right) OCR from *n* = 3 (OVCAR5), *n* = 5 (OVCAR8), and *n* = 2 (KURAMOCHI) independent experiments. A 2-tailed *t* test was used to calculate the *P* values (*P < 0.05, **P < 0.01). (**E**) Graphs show quantification of colony-forming assays for OVCAR5 and OVCAR8 untreated and MRD-mimic cells incubated with CPT1 inhibitors (see Methods). A 2-tailed *t* test was used to calculate the *P* values from *n* = 3 independent experiments.

Three different ovarian cancer cell lines (OVCAR5, OVCAR8, KURAMOCHI) were treated for 2 weeks with carboplatin concentrations that achieved more than 90% cell killing (end-of-treatment time point); then, the surviving cells were allowed to recover in normal medium (MRD-mimic time point) (Figure 6A), mimicking a scenario followed by HGSOCs in patients between NACT and IDS where sampling of MRD occurred.

Similar to what we observed in vivo, after carboplatin treatment the cells displayed a more elongated morphology as well as higher level of expression of mesenchymal markers (Supplemental Figure 6, A and B).

Moreover, the surviving cells at the end of treatment (Figure 6A) showed significant upregulation of several adipocyte signature genes that were also identified in MRD in patients, such as the fatty acid synthase *FASN*, the lipid droplet-associated protein perilipin 1 (*PLIN1*), and the peroxisome proliferator–activated receptor γ (*PPARG*) (Figure 6B).

Increased expression of other genes involved in lipid-related pathways was also observed, including *CPT1A*, *PPARA*, and *ACADM* (Figure 6B). These changes were maintained in MRD-mimic cells upon withdrawal of the carboplatin (Supplemental Figure 6C), suggesting that the resistant cells had a long-lasting phenotype that may be important for survival and tumor regeneration.

Next, we performed functional assays on the in vitro model to test whether lipid metabolism pathways were indeed perturbed in the cells that survived the carboplatin treatment. We used the Seahorse assay to measure mitochondrial oxygen consumption rate (OCR) as a readout of oxidative phosphorylation (OXPHOS). The MRD-mimic cells of all the tested lines showed a significantly higher OCR compared with the untreated cells, at both basal and maximal uncoupled states (Figure 6, C and D).

Similar results were also obtained at the end-of-treatment time point compared with untreated cells (Supplemental Figure 6, D and E), indicating that OXPHOS plays an important role in chemotherapy-resistant cells.

To elucidate which substrates are key for this process and evaluate a potential role of fatty acid (FA) in the survival of MRD-mimic cancer cells, we first blocked fatty acid oxidation (FAO) using etomoxir, an inhibitor of the carnitine palmitoyl transferase (*CPT1*) that imports FA into mitochondria for β-oxidation (25). Colony-forming assays were performed using OVCAR5 and OVCAR8 cells treated with etomoxir concentrations previously shown not to elicit off-target effects (26).

This approach revealed that the MRD-mimic cells of both cell lines displayed a significantly higher sensitivity than their carboplatin-untreated counterparts (Figure 6E and Supplemental Figure 6F). The results were confirmed using perhexiline, another CPT1 inhibitor currently used in the clinic as a prophylactic antianginal agent (27) (Figure 6E and Supplemental Figure 6F), showing that FAO is indeed crucial for chemotherapy-resistant cells, as already suggested by the transcriptome analyses described above.

Given that the lipid signature observed both in vivo and in vitro included not only genes related to FAO but also FA synthesis, we performed mass spectrometry-based lipidomics (28) to determine if the transcriptional increase of genes involved in de novo lipogenesis is reflected in an increase of storage lipids such as triacylglycerols (TAGs). Interestingly, for both OVCAR5 and OVCAR8 cell lines, the total concentration of glycerolipids, which comprises both TAGs and their immediate precursor diacylglcyerols, did not differ significantly before and after treatment with carboplatin (Supplemental Figure 6G). This suggests that, unlike FAO, the transcriptional changes observed in the FA synthesis pathway do not lead to an increase in lipid storage or that the newly synthesized FAs are immediately oxidized and therefore do not accumulate, as shown in the lipidome analysis.

Taken together these data confirm the robustness of our in vitro model and highlight that FAO is required for survival by MRD-mimic cancer cells, thus uncovering a new therapeutic vulnerability of MRD.

### FAO is a general mechanism of resistance in MRD that is independent of the cytotoxic agent.

Both our in vivo and in vitro data show that the ovarian cancer cells that survive DNA-targeting cytotoxic (carboplatin) or microtubule-stabilizing (paclitaxel) chemotherapy treatment are characterized by a transcriptional upregulation of their lipid metabolic pathways that appears to be a survival mechanism in MRD cancer cells. However, whether these observations represent a general survival mechanism under cytotoxic treatment or are more specifically related to the chemotherapeutics used remained unclear.

To investigate if the upregulation of lipid metabolism was a general survival mechanism in ovarian cancer cells, we tested the effect of poly-ADP ribose polymerase (PARP) inhibitor treatment on ovarian cancer cell lines. We selected this cytotoxic agent because its use is rapidly becoming standard of practice in patients with HGSOC, due to the defects in the homologous recombination repair pathway often found in these tumors (29). Moreover, it is known that PARP activation decreases the concentration of nicotinamide adenine dinucleotide, and this has been linked to lipid accumulation (30).

**Figure 7:**
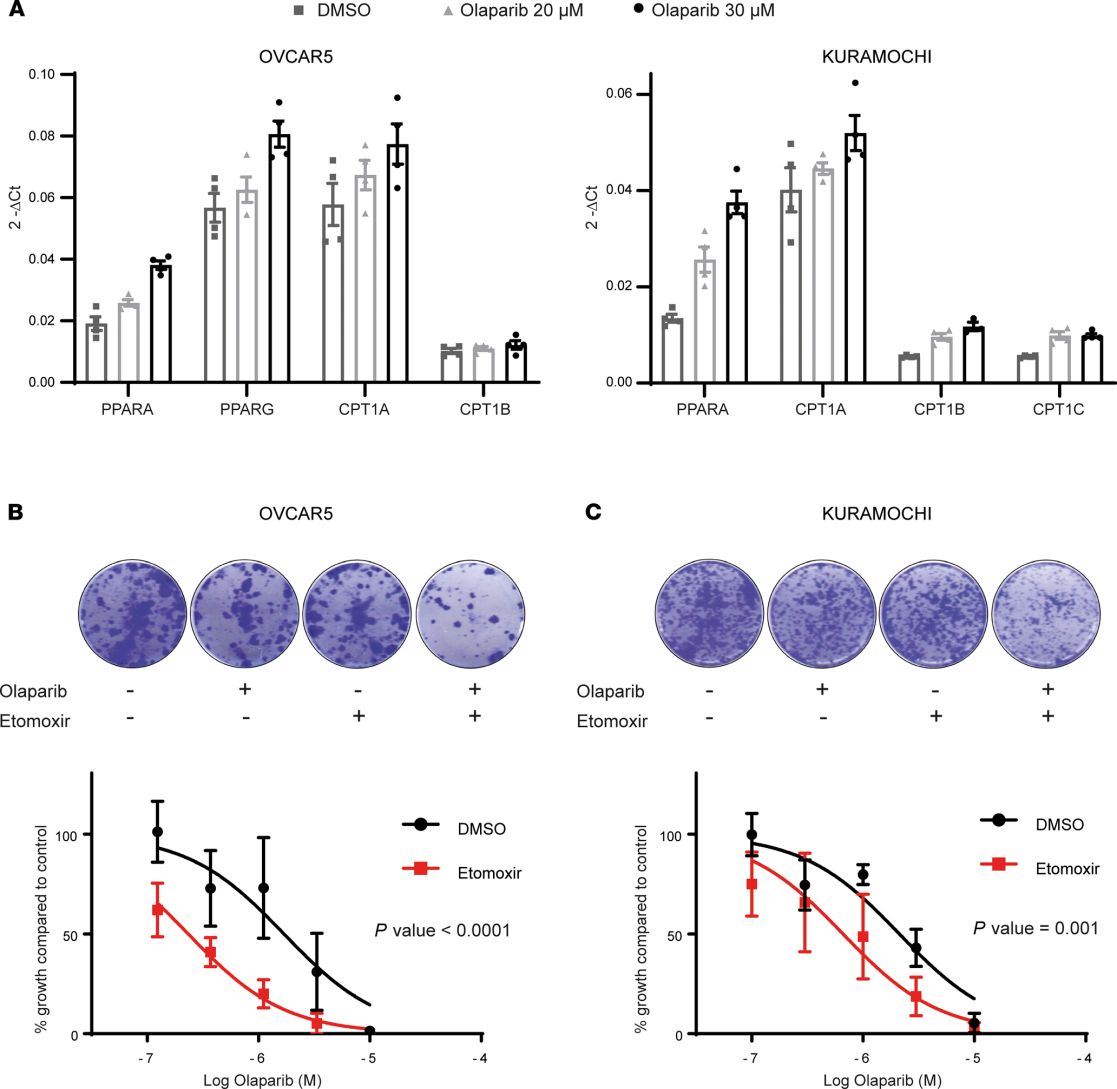
Inhibiting FAO enhances the cytotoxic effects of olaparib. (**A**) Quantitative real-time PCR of lipid metabolism genes in OVCAR5 (left) and KURAMOCHI (right) cells treated with different concentrations of the PARP inhibitor olaparib. The graph represents 2^-ΔCt^ of 4 technical replicates from *n* = 1. (**B**) Representative images from colony-forming assays of OVCAR5 cells treated with olaparib and 40 μM etomoxir (upper panel). Graph shows dose response to olaparib treatment with and without etomoxir (lower panel). A comparison of fits (*F* test) was performed on *n* = 3 independent experiments. (**C**) Representative images from colony-forming assays of KURAMOCHI cells treated with olaparib and 40 μM etomoxir (upper panel). Graph shows dose response to olaparib treatment with and without etomoxir (lower panel). A comparison of fits (*F* test) was performed on *n* = 3 independent experiments.

First, we measured the expression of key lipid metabolism genes using quantitative PCR following olaparib treatment. KURAMOCHI and OVCAR5 cells showed an upregulation of *PPARA* and genes belonging to the *CPT* family at both concentrations of olaparib that were tested (Figure 7A). In hepatocyte, adipocyte, and myoblast cells, it was reported that PARP inhibitors activate the expression of FAO-related genes through Sirtuin1 (SIRT1) activation (31, 32); however, in our system, SIRT1 knockdown did not change the upregulation of FAO genes upon treatment with olaparib (Supplemental Figure 7A).

Next, we examined if the inhibition of FAO had any effect on the sensitivity of ovarian cancer cells to olaparib. The colony formation ability for both KURAMOCHI and OVCAR5 lines was significantly reduced when the cells were treated with a combination of etomoxir and olaparib compared with single treatment (Figure 7, B and C). Similar results were also obtained for OVCAR8 and SKOv3 cell lines (Supplemental Figure 7B).

These findings indicate that the upregulation of lipid metabolism may be a more general mechanism through which ovarian cancer cells survive cytotoxic stress as it is not restricted to any particular type of chemotherapeutic treatment. Furthermore, inhibiting FAO may represent a therapeutic strategy to enhance the efficacy of cytotoxic treatment in HGSOC.

Moreover, these findings suggest that preventing cells from entering into an adipocyte-like cell state could represent a new therapeutic strategy to sensitize ovarian cancer cells to cytotoxic treatment.

## Discussion

Treatment of MRD in solid tumors requires a better understanding of the mechanisms of survival of cancer cells that remain at the end of treatment. However, this has been hampered by the difficulties in selecting representative sites from which to sample MRD, the invasive nature of the sampling techniques required, and the challenges of the molecular characterization of minute amounts of material from clinical samples. Therefore, most of the knowledge base of MRD in solid tumors is derived from analyzing preclinical models. In this study, we have designed a clinical trial to specifically address these issues and successfully obtained a pure collection of MRD samples that enabled us to gain informative insights about MRD biology in ovarian cancer patients. Our analysis revealed a potentially previously unrecognized adipocyte-like signature in MRD in HGSOC. We have complemented our in vivo approach with validation in an in vitro MRD-mimic model that we developed. Using this model, we demonstrate that MRD upregulated FAO and that the specific inhibition of this process synergized with chemotherapeutics, increasing their cancer cell killing potential. We show that upregulation of FAO seems to be a general survival mechanism in MRD following chemotherapy or PARP inhibition, and thus, despite the small number of patients analyzed and the limited mechanistic studies, our results have important therapeutic implications.

To the best of our knowledge, this work represents the first comprehensive characterization of MRD from HGSOC clinical samples. Through an LCM-guided RNA-Seq approach, we demonstrate that these microscopic tumor foci, isolated from exceptional responders after NACT, not only had features of TICs but also showed altered lipid metabolism that has clinical relevance. Alongside a marked EMT phenotype and the upregulation of several genes belonging to the ABC and ALDH families, these cells had increased expression of the desaturase *SCD*, which is consistent with previous observations that ovarian TICs have high levels of unsaturated lipids (33). The identification of several transcriptomic features that are typical of TICs strongly supports the hypothesis that MRD in the peritoneal cavity is indeed responsible for relapse. Targeting these residual chemotherapy-resistant cells would therefore be highly promising. Our data suggest that targeting lipid metabolism could represent an attractive therapeutic option, similar to what has been observed in vitro or in preclinical models of other cancer types (34–37).

Using our deconvolution-based classifier (22, 23), we have shown that MRD from the exceptional responders is enriched in EMT-high cancer cells. The EMT process is known to facilitate tumor progression. For example, several mechanisms through which EMT induces stemness have now been elucidated (38). Moreover, metabolic reprogramming has been associated with EMT plasticity (39), and TGF-β1–induced mesenchymal cells display a shift from glycolysis to OXPHOS (40). A similar metabolic shift has been previously described in breast (41) and pancreatic (42) cancers using mouse models of oncogenic pathway inhibition to mimic MRD. However, the transcriptomics changes described in those studies were not as extreme as the adipocyte-like signature observed here, possibly due to differences across species or the specific organ tropism of ovarian cancer.

The perturbation of lipid metabolism observed in MRD can be explained by at least 2 models that are not necessarily mutually exclusive. The first one is that a subpopulation of cells in the primary tumor already expresses the adipocyte-like gene signature and that these cells become selected upon treatment because such altered metabolism confers a survival advantage for MRD. Given the EMT enrichment in MRD, one might argue that the adipocyte-like gene signature is an inherent feature of the EMT-high cancer cell state. Through the analysis of publicly available data sets of prechemotherapy HGSOC, we have shown that this might indeed be the case, since many genes from the adipocyte-like signature showed significantly higher expression in EMT-high tumors. Additional evidence supporting this idea of a selection process is provided by the observation that primary prechemotherapy HGSOC displays OXPHOS metabolic heterogeneity: the high-OXPHOS group exhibits better short-term survival because its chronic oxidative stress makes it more sensitive to chemotherapy (43). This is consistent with the initial good clinical response observed in the exceptional responders; however, we would argue that the high-OXPHOS cells that survive treatment may eventually lead to recurrence because they have found mechanisms to reduce oxidative stress, such as the activation of a temporary dormant state.

An alternative hypothesis is that cells with altered regulation of lipid metabolism are absent before chemotherapy and that this metabolic rewiring occurs in response to chemotherapy. Some degree of chemotherapy induction cannot be excluded from our data, and detailed time-lapse metabolic analysis will be needed to further investigate this possibility.

Irrespective of how this adipocyte-like signature becomes so preponderant in MRD (whether it is by selection or induction), the implications for treatment remain clear and suggest that the inclusion of therapeutics targeting FAO may be beneficial for HGSOC patients. The successful inhibition of CPT1, for example, could represent a new therapeutic approach to sensitize ovarian cancer cells to different cytotoxic treatments, such as carboplatin and olaparib. In addition, our work clearly shows that the MRD cells have marked mesenchymal characteristics. It is now widely recognized that EMT causes resistance to several anticancer agents, spanning from chemotherapy to immunotherapy. All research efforts to tackle EMT-induced multidrug resistance have so far focused on strategies to prevent or reverse EMT (44), and only recently the significant metabolic rewiring associated with EMT has started to gain attention as a potential therapeutic target (45). Our findings provide new insights in this ongoing debate and suggest an alternative way forward to overcome EMT-related resistance, at least in MRD.

In conclusion, we suggest that targeting FAO may be an attractive strategy to eradicate MRD in HGSOC and improve the long-term survival of exceptional responders.

## Methods

### Cell lines and cell culture.

Cell lines were obtained from American Type Culture Collection (ATCC) (OVCAR5, OVCAR8, SKOv3, 3T3-L1) and the Japanese Collection of Research Bioresources Cell Bank (KURAMOCHI).

SKOv3 cells were cultured in McCoy’s 5A (Gibco, Thermo Fisher Scientific); OVCAR5, OVCAR8, and KURAMOCHI cells in RPMI 1640 (Gibco, Thermo Fisher Scientific) with fetal bovine serum (10%; Gibco, Thermo Fisher Scientific) and penicillin-streptomycin (100 U; Gibco, Thermo Fisher Scientific); and 3T3-L1 cells in DMEM (Thermo Fisher Scientific) containing 10% calf serum (MilliporeSigma). All lines were kept at 37°C, 5% CO_2_, and 95% humidity.

### Tumor samples.

Tumor samples were biopsied during diagnostic laparoscopy or IDS, immediately frozen on dry ice, and stored in clearly labeled cryovials in –80°C freezers.

### Sample processing and sectioning for LCM.

Frozen tumor samples were embedded in OCT (NEG-50, Richard-Allan Scientific), and 10 μm sections were taken using MB DynaSharp microtome blades (Thermo Fisher Scientific) in a CryoStar cryostat microtome (Thermo Fisher Scientific). The first tissue section was mounted onto regular glass slides (SuperFrost Plus, VWR International) for hematoxylin (Hematoxylin solution, Gill No. 3, MilliporeSigma) and eosin (Eosin Y solution, MilliporeSigma) staining (H&E), according to the manufacturer’s instructions, followed by 6 to 10 sequential tissue sections onto polyethylene naphthalate membrane (PEN) glass slides (MembraneSlide 1.0 PEN, Zeiss), which were immediately stained on ice (2 minutes in 70% ethanol, 2 minutes in 1% Cresyl violet from MilliporeSigma in 50% ethanol, followed by rinse in 100% ethanol), then stored at –80°C. Nuclease-free technique was used throughout the procedure. After each H&E the slide was reviewed by a gynecological oncology pathologist to confirm the presence of cancer cells and delineate their location. A PALM Laser Microdissection System (Zeiss) was used to catapult individual tumor islets into a 200 μL opaque AdhesiveCap (Zeiss). Images of target area in 5× and 10× original magnification as well as of caps with captured material were obtained for documentation. For the “prechemo” samples and the “postchemo” sites where there was evidence of poor response, scrolls were also collected to be used for bulk RNA-Seq.

### RNA extraction and library preparation.

Both laser capture microdissected tumor islets and bulk scrolls were immediately processed for RNA extraction using the RNAqueous-Micro Total RNA Isolation Kit (Thermo Fisher Scientific) and the RNeasy Mini Kit (QIAGEN), respectively. Both extraction procedures were performed according to the manufacturer’s instructions, including DNase digestion, after which RNA integrity was evaluated using the 2200 TapeStation (Agilent). The SMARTer Stranded Total RNA-seq kit v2 — Pico Input (Takara) was used to prepare sequencing libraries from LCM material and the KAPA mRNA HyperPrep Kits (Roche) for the bulk scrolls.

All libraries were assessed with TapeStation (Agilent) and then quantified by Qubit (Thermo Fisher Scientific). Multiplexed library pools were quantified with the KAPA Library Quantification Kit (Roche) and sequenced using 75 bp paired-end reads on the NextSeq500 platform (Illumina).

### Adipocyte differentiation.

3T3-L1 preadipocytes were treated as previously described (17).

### In vitro MRD model.

Carboplatin (Cambridge Bioscience) concentrations were optimized for each cell line in order to obtain more than 90% cell killing after a 2-week treatment (5 μg/mL for KURAMOCHI, 3 μg/mL for OVCAR5, 2 μg/mL for OVCAR8). RNA was extracted using the RNAqueous-Micro Total RNA Isolation Kit (Thermo Fisher Scientific) and retro-transcribed with TaqMan Reverse Transcription Reagents (Thermo Fisher Scientific). All the quantitative real-time PCR experiments were performed on the CFX Bio-Rad system using SYBR Green PCR Master Mix (Thermo Fisher Scientific).

### OCR analysis.

Cells were seeded in XFe96 Cell Culture Microplates (Seahorse) at 70%–80% confluence and incubated at 37°C in 5% CO_2_ atmosphere. After 24 hours OCR was measured on the XF96 Analyzer (Agilent) using the Seahorse XF Cell Mito Stress Test Kit (Agilent) according to the manufacturer’s instructions.

### Colony-forming assays.

Cells from the MRD in vitro model were plated on 12-well plates and treated with etomoxir (40 μM), perhexiline (2 μM), or DMSO as a control. Cells from the PARP inhibition experiment were plated on 12-well plates and treated with etomoxir (40 μM for OVCAR5 and KURAMOCHI, 60 μM for OVCAR8, 80 μM for SKOv3), olaparib (0.1–10 μM for SKOv3, OVCAR5, KURAMOCHI, 0.01–1 μM for OVCAR8), or the combination of the 2 drugs.

After 2 weeks all cells were washed with PBS, fixed with cold methanol, and stained with crystal violet solution (0.5 g CV in MilliQ water/20% methanol) for 30 minutes, followed by washing. Plates were scanned and individual colonies were counted. For OVCAR5 cells, which do not grow in discrete individual colonies, a relative measure of cell number was instead determined by solubilizing the staining with 10% acetic acid, then measuring the absorbance at 590 nm.

### Transfections.

Transient knockdown experiments were performed by transfection with a validated nontargeting siRNA or SIRT1 siRNA (SMARTpool, ON-TARGETplus, Horizon Discovery), using Dhermafect 4 transfection reagent (Horizon Discovery) according to the manufacturer’s instructions. Cells were reverse-transfected with siRNAs for up to 72 hours before they were harvested to obtain RNA for quantitative real-time PCR experiments.

### Mass spectrometry–based lipidomics.

Cells were harvested in HKM buffer (50 mM HEPES, 50 mM KOH, 150 mM KCl, 5 mM MgCl_2_, pH 7.5), and lipidomics analysis was performed as previously described (17, 28, 46).

### Preprocessing of RNA-Seq data.

Sequencing reads from FASTQ files were trimmed for adapter sequences and quality with Trim Galore!, mapped to the UCSC hg19 human genome assembly using STAR (v2.4.2a), and read counts were obtained using subread FeatureCounts (v1.4.5-p1).

### Pseudotime analysis.

We used the R package PhenoPath (11) to perform the pseudotime analysis that projected the high-dimensional transcriptomic data to 1 dimension, in which we compared the OXO-PCR cohort and the TCGA data set.

### Differential expression analysis.

Differential expression analysis was carried out using edgeR (v3.10.5).

### Biological process and reactome enrichment analysis.

The statistical overrepresentation was performed with PANTHER (v14.1), and the threshold for significance was set at FDR < 0.05.

### Deconvolution of OXO-PCR RNA-Seq data.

To estimate the proportions of 5 previously identified molecular signatures (differentiated, KRT17 cluster, EMT, cell cycle, and ciliated), we used the deconvolution algorithm, CIBERSORT (47), and the reference matrix derived from single-cell RNA-Seq data of human fallopian tubes in our previous work (22). The deconvolution analysis was applied on OXO-PCR samples of over 100,000 read counts. The CIBERSORT R script v1.04 (last updated October 24, 2016) was downloaded from the CIBERSORT website (https://cibersort.stanford.edu) and run locally in R v3.6.0. The proportions of 5 molecular signatures, i.e., scores, were calculated by applying the linear support vector regression, which was incorporated in the CIBERSORT function, on the raw expression profiles of each tumor sample. The deconvolution analysis was performed in the relative mode, and thus, for each tumor the scores of 5 molecular signatures added up to 1.

### Analysis of TCGA and AOCS data.

TCGA Illumina HiSeq UNC RNA-Seq data set (version: 2017-10-13) was downloaded from the UCSC Xena Data Hub (https://tcga.xenahubs.net) (12, 48). The AOCS data set was downloaded from GSE9899 (24). TCGA and AOCS data were transferred to a non–log-linear space and then deconvolved in the same way as the OXO-PCR RNA-Seq data. Samples were partitioned into 3 groups, EMT-low, -middle, and -high groups, for each data set. We compared the expression of 5 genes related to lipid metabolism between EMT-high and EMT-low samples by using differential expression analysis (limma voom) (49).

### Data availability.

The bulk and LCM RNA-Seq data sets are deposited at Gene Expression Omnibus (accession numbers GSE132107 and GSE162714).

### Statistics.

Data were analyzed for statistical difference using 2-tailed unpaired *t* test for 2-group comparisons (GraphPad Prism 9.0.1). Statistical significance was defined as a *P* value of less than 0.05.

### Study approval.

The cases in this study were recruited under the Gynaecological Oncology Targeted Therapy Study 01 (GO-Target-01, NHS Health Research Authority South Central – Berkshire Research Ethics Committee research ethics approval 11-SC-0014) and the Oxford Ovarian Cancer Predict Chemotherapy Response Trial (OXO-PCR-01, NHS Health Research Authority South Central – Berkshire Research Ethics Committee research ethics approval 12-SC-0404). All participants involved in this study were appropriately informed and consented.

## Author contributions

AAA, TSS, RCB, and MA conceived, administered, and supervised the project; AAA, TSS, RCB, and ZL acquired funding; MA, CY, ZH, KM, PCR, GM, NW, MM, KC, MN, CEZ, SES, LC, TM, and A Alsaadi investigated; MA, AAA, TSS, CY, ALH, RWK, CSE, ZH, KM, PCR, GM, NW, MM, FG, KC, MN, CEZ, SES, SPB, TM, A Albukhari, A Alsaadi, and LSG discussed and interpreted results; MA, AAA, CY, and ZH curated data; MA, AAA, CY, ZH, RWK, CSE, and PCR developed methodology and performed formal analysis and visualization; MA, AAA, and A Albukhari wrote the original draft; AAA, TSS, and MA reviewed and edited the draft; and MM, S Damato, S Dhar, NW, PNP, SN, and AL provided clinical samples.

## Supplementary Material

Supplemental data

Supplemental Table 1

Supplemental Table 2

Supplemental Table 3

Supplemental Table 4

Supplemental Video 1

## Version 1 05/04/2021

In-Press Preview

## Version 2 06/08/2021

Electronic publication
