# Supplementary material for: Adipocyte-like signature in ovarian cancer minimal residual disease identifies metabolic vulnerabilities of tumor-initiating cells
Source: JCI Insight. 2021 Jun 8;6(11):e147929. doi: 10.1172/jci.insight.147929 (PMC8262282; doi:10.1172/jci.insight.147929)
Supplement: Supplemental data [file jciinsight-6-147929-s099.pdf]

Supplementary Figures

Supplementary Figure 1. The Oxford Ovarian Cancer Predict Chemotherapy Response (OXO-PCR) study: a patient cohort.

(A) Diagram shows the LCM and bulk RNA-seq pipelines including the multiple quality control steps to avoid the contamination from surrounding non-cancer tissue.

(B) LCM images of the 6 MRD samples captured from the Exceptional Responders (n=3). Scale bars, 50 µm.

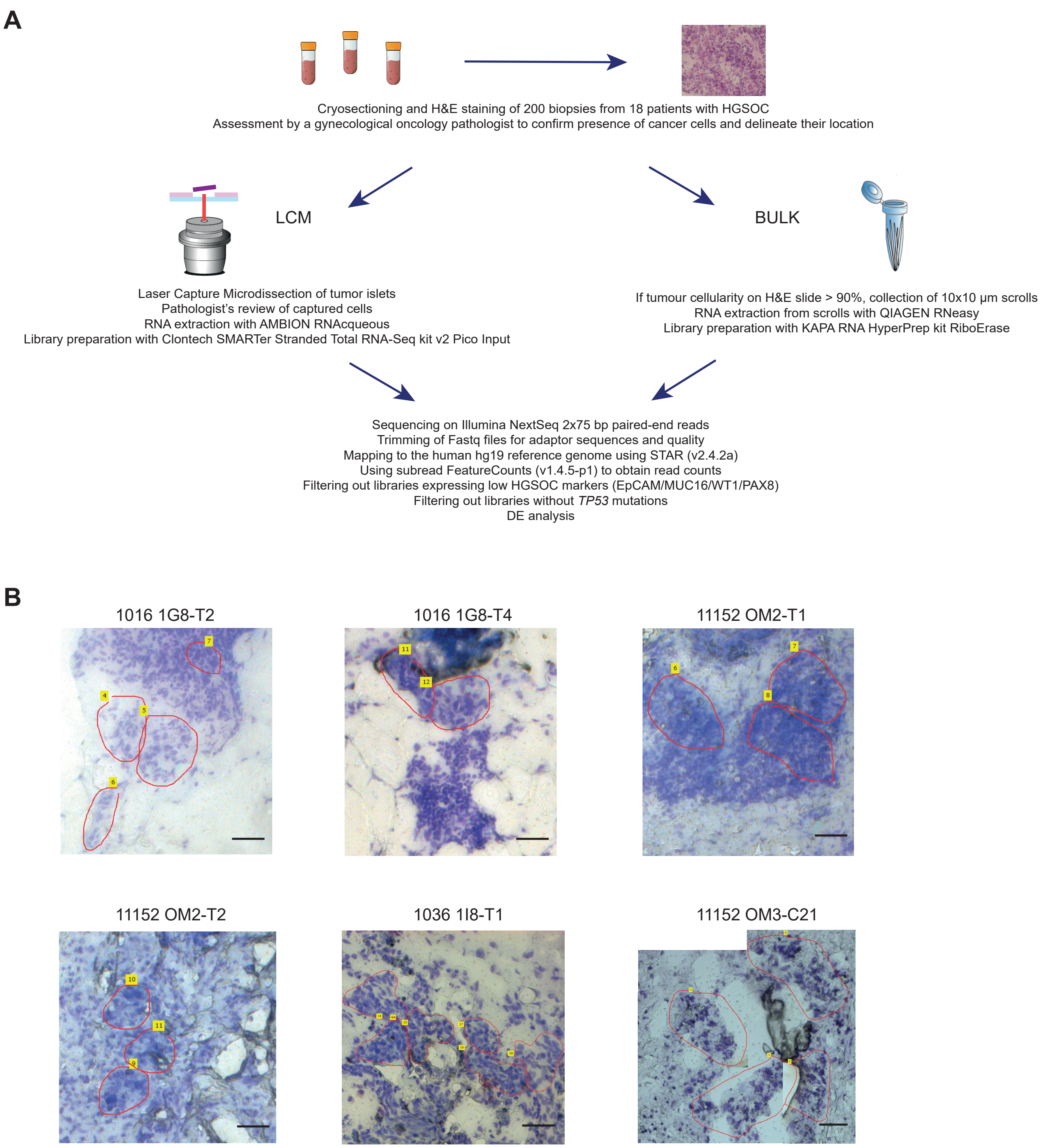
A
Cryosectioning and H&E staining of 200 biopsies from 18 patients with HGSOC
Assessment by a gynecological oncology pathologist to confirm presence of cancer cells and delineate their location
LCM
BULK
Laser Capture Microdissection of tumor islets
Pathologist's review of captured cells
RNA extraction with AMBION RNAcqueous
Library preparation with Clontech SMARTer Stranded Total RNA-Seq kit v2 Pico Input
If tumour cellularity on H&E slide > 90%, collection of 10x10 μm scrolls
RNA extraction from scrolls with QIAGEN RNeasy
Library preparation with KAPA RNA HyperPrep kit RiboErase
Sequencing on Illumina NextSeq 2x75 bp paired-end reads
Trimming of Fastq files for adaptor sequences and quality
Mapping to the human hg19 reference genome using STAR (v2.4.2a)
Using subread FeatureCounts (v1.4.5-p1) to obtain read counts
Filtering out libraries expressing low HGSOC markers (EpCAM/MUC16/WT1/PAX8)
Filtering out libraries without TP53 mutations
DE analysis
B
1016 1G8-T2
1016 1G8-T4
11152 OM2-T1
11152 OM2-T2
1036 1I8-T1
11152 OM3-C21

Supplementary Figure 2. Pseudotime and t-SNE analysis reveal limited intra-patient heterogeneity.

(A) Plot shows the unsupervised pseudotime analysis of the OXO-PCR pre-chemotherapy sample set and the TCGA HGSOC cohort.

(B) t-SNE plot of the entire OXO-PCR dataset following batch correction.

(C) Dot plot shows the main pathways enriched among the pseudotime-dependent genes. Differential expression analysis was performed along the pseudotime gradient independently of chemotherapy effect.

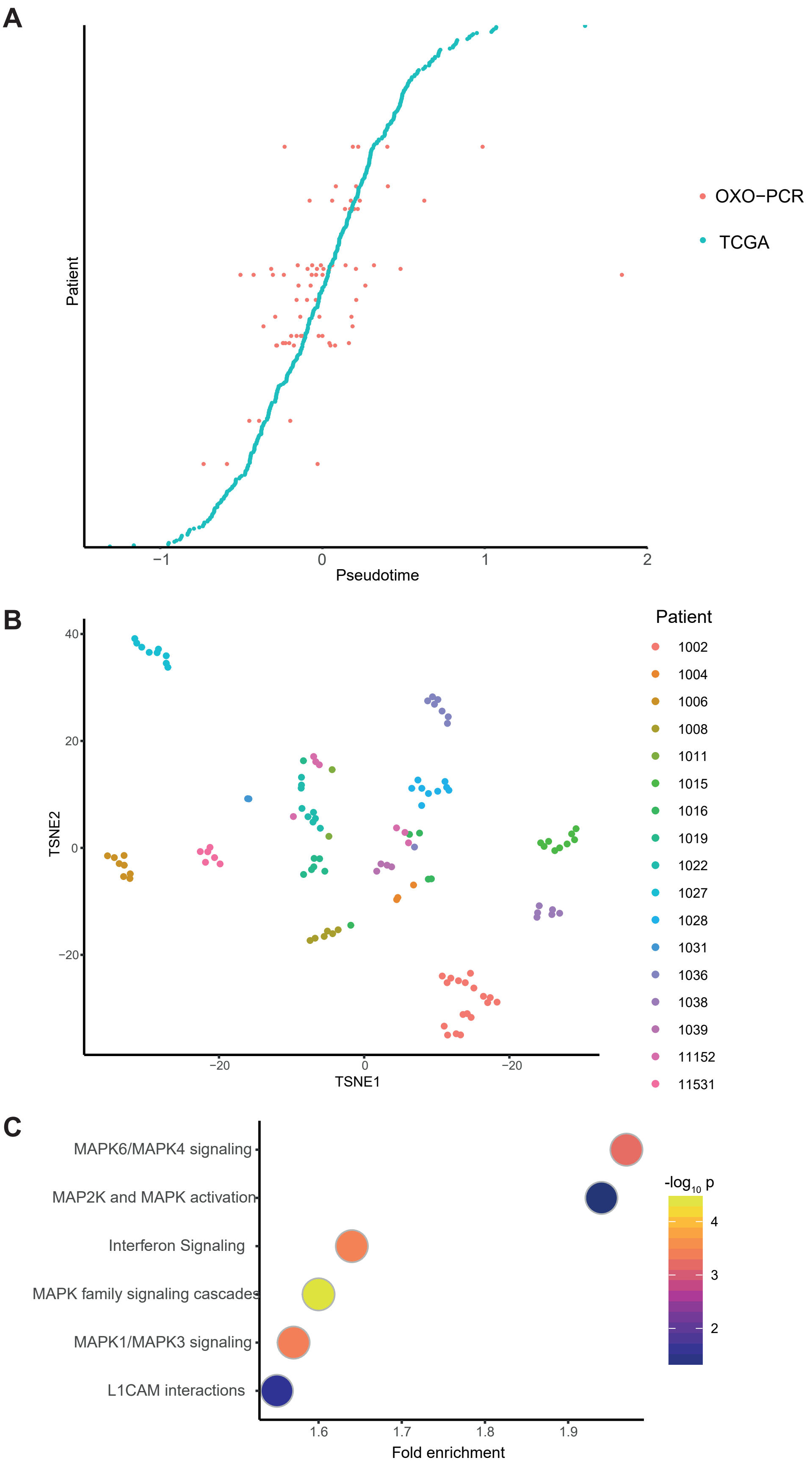
A
Patient
−1
0
1
2
Pseudotime
OXO−PCR
TCGA
B
Patient
1002
1004
1006
1008
1011
1015
1016
1019
1022
1027
1028
1031
1036
1038
1039
11152
11531
TSNE2
40
20
0
-20
TSNE1
−20
0
−20
C
MAPK6/MAPK4 signaling
MAP2K and MAPK activation
Interferon Signaling
MAPK family signaling cascades
MAPK1/MAPK3 signaling
L1CAM interactions
1.6
1.7
1.8
1.9
Fold enrichment
-log10 p
4
3
2

Supplementary Figure 3. HGSOC MRD cells exhibit adipocyte-like and TICs transcriptomic signatures.

(A) Dot plot shows the expression of HGSOC marker genes in the exceptional responders before and after chemotherapy.

(B) Heatmap shows three selections of differentially expressed genes for the exceptional responder patient 1016: lipid metabolism genes, ABC transporters and dormancy genes.

(C) Heatmap shows the lipid metabolism genes differentially expressed in the exceptional responder patient 1036.

(D) Graphs show expression levels of lipid metabolism genes in the exceptional responder patient 11152.

(E) Quantitative real-time PCR of two lipid metabolism genes in the exceptional responders before and after chemotherapy.

(F) Graphs show expression levels of genes from the adipocyte-like signature in poor and exceptional responders after treatment.

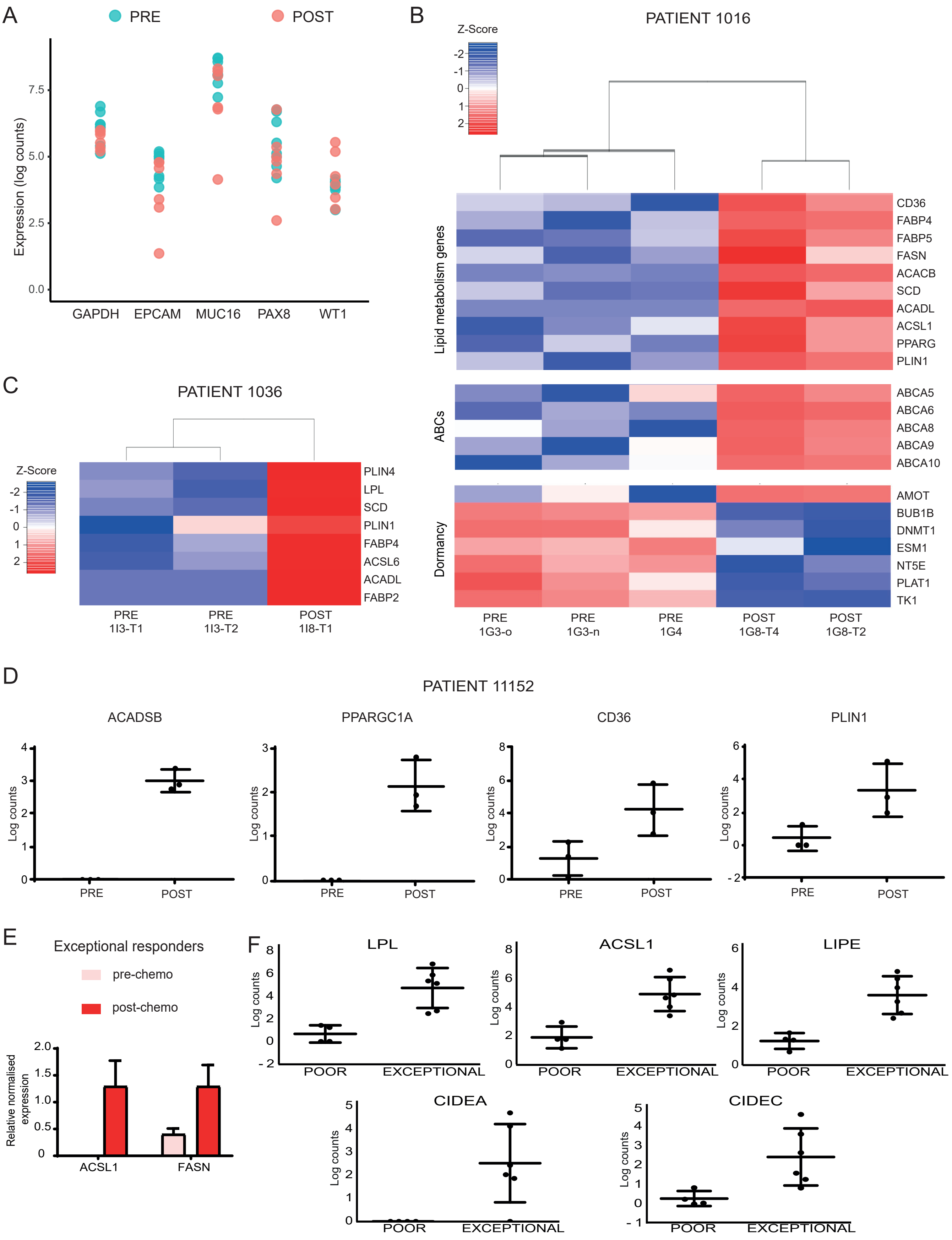

A
PRE
POST
Expression (log counts)
7.5
5.0
2.5
0.0
GAPDH
EPCAM
MUC16
PAX8
WT1
B
PATIENT 1016
Z-Score
-2
-1
0
1
2
Lipid metabolism genes
CD36
FABP4
FABP5
FASN
ACACB
SCD
ACADL
ACSL1
PPARG
PLIN1
ABCs
ABCA5
ABCA6
ABCA8
ABCA9
ABCA10
Dormancy
AMOT
BUB1B
DNMT1
ESM1
NT5E
PLAT1
TK1
PRE 1G3-o
PRE 1G3-n
PRE 1G4
POST 1G8-T4
POST 1G8-T2
C
PATIENT 1036
Z-Score
-2
-1
0
1
2
PLIN4
LPL
SCD
PLIN1
FABP4
ACSL6
ACADL
FABP2
PRE 1I3-T1
PRE 1I3-T2
POST 1I8-T1
D
PATIENT 11152
ACADSB
PPARGC1A
CD36
PLIN1
Log counts
PRE
POST
E
Exceptional responders
pre-chemo
post-chemo
Relative normalised expression
2.0
1.5
1.0
0.5
0
ACSL1
FASN
F
LPL
ACSL1
LIPE
CIDEA
CIDEC
Log counts
POOR
EXCEPTIONAL

Supplementary Figure 4. The transcriptome of MRD cells resembles differentiated adipocytes.

Bar plots show the transcriptional changes occurring during the differentiation of fibroblast-like 3T3-L1 cells into adipocytes.

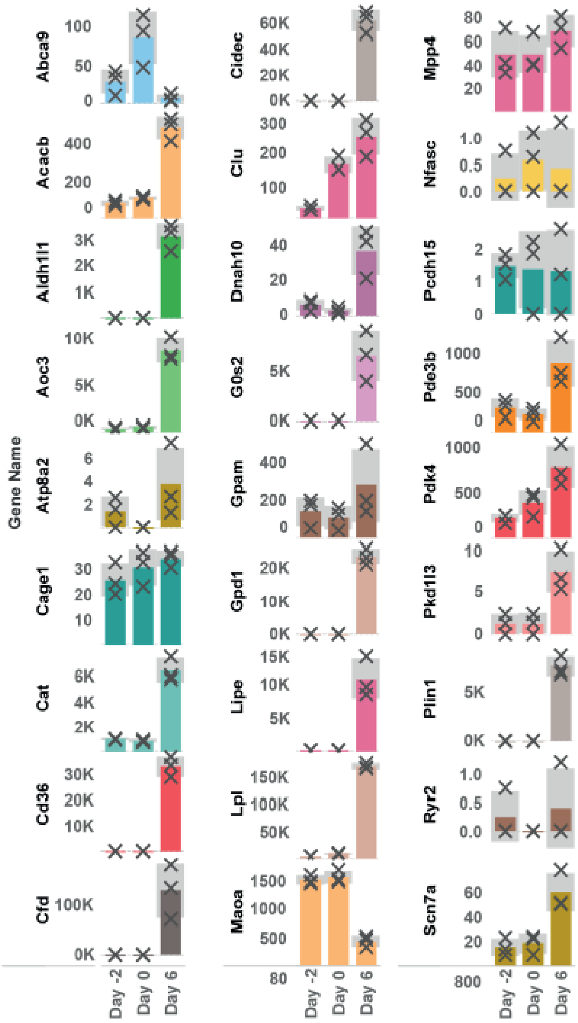
Gene Name
Abca9
Acacb
Aldh1l1
Aoc3
Atp8a2
Cage1
Cat
Cd36
Cfd
Cidec
Clu
Dnah10
G0s2
Gpam
Gpd1
Lipe
Lpl
Maoa
Mpp4
Nfasc
Pcdh15
Pde3b
Pdk4
Pkd1l3
Plin1
Ryr2
Scn7a
Day -2
Day 0
Day 6

Supplementary Figure 5. MRD cells show mesenchymal characteristics

Stacked bar plot visualizes the deconvolution result of the whole OXO-PCR dataset. Colors of the bars denote the 5 cell states as shown in the legend.

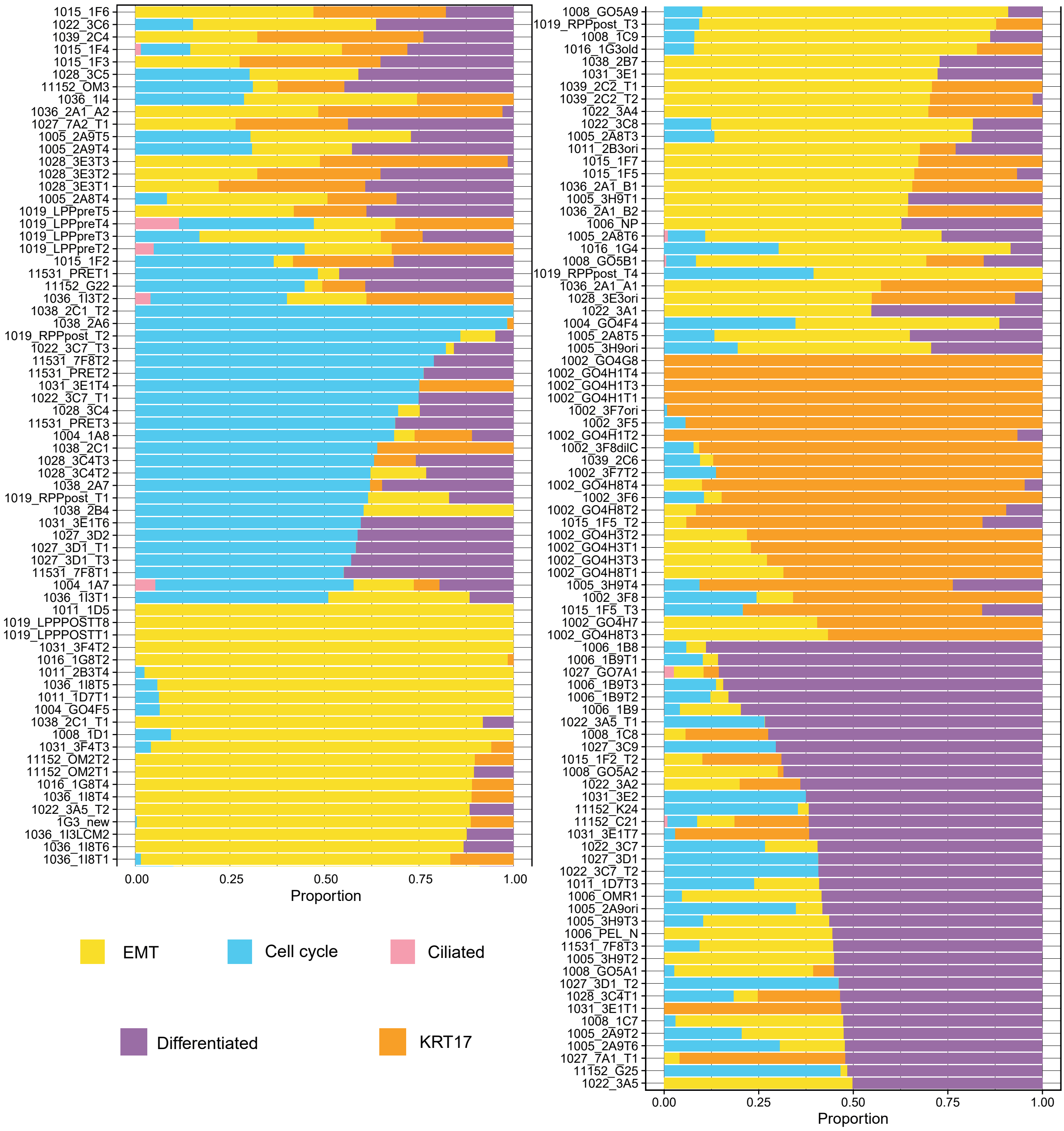
1015_1F6
1022_3C6
1039_2C4
1015_1F4
1015_1F3
1028_3C5
11152_OM3
1036_1I4
1036_2A1_A2
1027_7A2_T1
1005_2A9T5
1005_2A9T4
1028_3E3T3
1028_3E3T2
1028_3E3T1
1005_2A8T4
1019_LPPpreT5
1019_LPPpreT4
1019_LPPpreT3
1019_LPPpreT2
1015_1F2
11531_PRET1
11152_G22
1036_1I3T2
1038_2C1_T2
1038_2A6
1019_RPPpost_T2
1022_3C7_T3
11531_7F8T2
11531_PRET2
1031_3E1T4
1022_3C7_T1
1028_3C4
11531_PRET3
1004_1A8
1038_2C1
1028_3C4T3
1028_3C4T2
1038_2A7
1019_RPPpost_T1
1038_2B4
1031_3E1T6
1027_3D2
1027_3D1_T1
1027_3D1_T3
11531_7F8T1
1004_1A7
1036_1I3T1
1011_1D5
1019_LPPPOSTT8
1019_LPPPOSTT1
1031_3F4T2
1016_1G8T2
1011_2B3T4
1036_1I8T5
1011_1D7T1
1004_GO4F5
1038_2C1_T1
1008_1D1
1031_3F4T3
11152_OM2T2
11152_OM2T1
1016_1G8T4
1036_1I8T4
1022_3A5_T2
1G3_new
1036_1I3LCM2
1036_1I8T6
1036_1I8T1
0.00
0.25
0.50
0.75
1.00
Proportion
EMT
Cell cycle
Ciliated
Differentiated
KRT17
1008_GO5A9
1019_RPPpost_T3
1008_1C9
1016_1G3old
1038_2B7
1031_3E1
1039_2C2_T1
1039_2C2_T2
1022_3A4
1022_3C8
1005_2A8T3
1011_2B3ori
1015_1F7
1015_1F5
1036_2A1_B1
1005_3H9T1
1036_2A1_B2
1006_NP
1005_2A8T6
1016_1G4
1008_GO5B1
1019_RPPpost_T4
1036_2A1_A1
1028_3E3ori
1022_3A1
1004_GO4F4
1005_2A8T5
1005_3H9ori
1002_GO4G8
1002_GO4H1T4
1002_GO4H1T3
1002_GO4H1T1
1002_3F7ori
1002_3F5
1002_GO4H1T2
1002_3F8dilC
1039_2C6
1002_3F7T2
1002_GO4H8T4
1002_3F6
1002_GO4H8T2
1015_1F5_T2
1002_GO4H3T2
1002_GO4H3T1
1002_GO4H3T3
1002_GO4H8T1
1005_3H9T4
1002_3F8
1015_1F5_T3
1002_GO4H7
1002_GO4H8T3
1006_1B8
1006_1B9T1
1027_GO7A1
1006_1B9T3
1006_1B9T2
1006_1B9
1022_3A5_T1
1008_1C8
1027_3C9
1015_1F2_T2
1008_GO5A2
1022_3A2
1031_3E2
11152_K24
11152_C21
1031_3E1T7
1022_3C7
1027_3D1
1022_3C7_T2
1011_1D7T3
1006_OMR1
1005_2A9ori
1005_3H9T3
1006_PEL_N
11531_7F8T3
1005_3H9T2
1008_GO5A1
1027_3D1_T2
1028_3C4T1
1031_3E1T1
1008_1C7
1005_2A9T2
1005_2A9T6
1027_7A1_T1
11152_G25
1022_3A5
0.00
0.25
0.50
0.75
1.00
Proportion

Supplementary Figure 6. MRD-mimic cells in vitro are sensitive to inhibitors targeting fatty acid oxidation.

(A) Representative images show OVCAR5 and OVCAR8 cells before and after carboplatin treatment.

(B) Quantitative real-time PCR of three EMT drivers in KURAMOCHI, OVCAR5 and OVCAR8 cells before and after carboplatin treatment.

(C) Quantitative real-time PCR of PPARA and FASN mRNA in OVCAR5 MRD-mimic cells recovered in regular medium for different amounts of time.

(D-E) Representative pattern of OCR as a function of time (min) normalized to DNA content in untreated, end of treatment and MRD-mimic OVCAR5 cells (D). Bar plots show means ± SEM basal (top) and maximal (bottom) OCR from 3 independent experiments.

(F) Representative images of colony forming assays for OVCAR5 and OVCAR8 untreated and MRD-mimic cells incubated with CPT1 inhibitors (see methods).

(G) Bar plots show lipid concentration and composition in OVCAR5 and OVCAR8 cell lines before and after treatment with carboplatin.

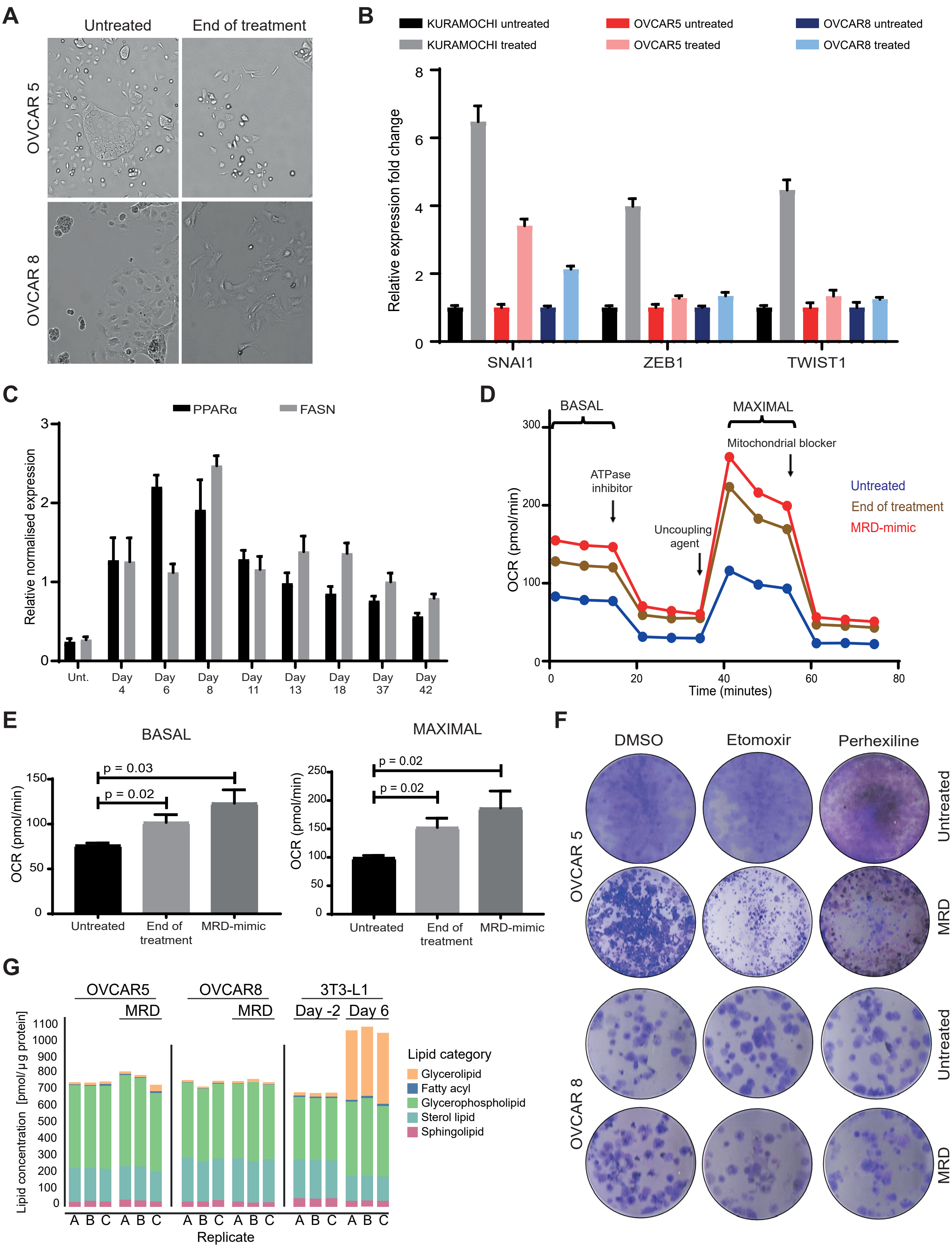

A
Untreated
End of treatment
OVCAR 5
OVCAR 8
B
KURAMOCHI untreated
KURAMOCHI treated
OVCAR5 untreated
OVCAR5 treated
OVCAR8 untreated
OVCAR8 treated
Relative expression fold change
SNAI1
ZEB1
TWIST1
C
PPARα
FASN
Relative normalised expression
Unt.
Day 4
Day 6
Day 8
Day 11
Day 13
Day 18
Day 37
Day 42
D
BASAL
MAXIMAL
ATPase inhibitor
Uncoupling agent
Mitochondrial blocker
Untreated
End of treatment
MRD-mimic
OCR (pmol/min)
Time (minutes)
E
BASAL
MAXIMAL
p = 0.03
p = 0.02
p = 0.02
p = 0.02
OCR (pmol/min)
Untreated
End of treatment
MRD-mimic
F
DMSO
Etomoxir
Perhexiline
OVCAR 5
OVCAR 8
Untreated
MRD
G
OVCAR5
OVCAR8
3T3-L1
MRD
Day -2
Day 6
Lipid concentration [pmol/μg protein]
Lipid category
Glycerolipid
Fatty acyl
Glycerophospholipid
Sterol lipid
Sphingolipid
A B C A B C
Replicate

Supplementary Figure 7. Fatty acid oxidation is a general mechanism of resistance in MRD that is independent from the cytotoxic agent.

(A) Quantitative real-time PCR of FAO genes in KURAMOCHI cells with SIRT1 knockdown upon olaparib treatment. The graph represents fold change of mean expression relative to siNonTargeting DMSO controls from n=1.

(B) Graphs show dose response to olaparib treatment with and without etomoxir in colony forming assays. SKOV3 cells were treated with 80 μM etomoxir and OVCAR8 with 60 μM etomoxir. A comparison of fits (F test) was performed on n=3 independent experiments.

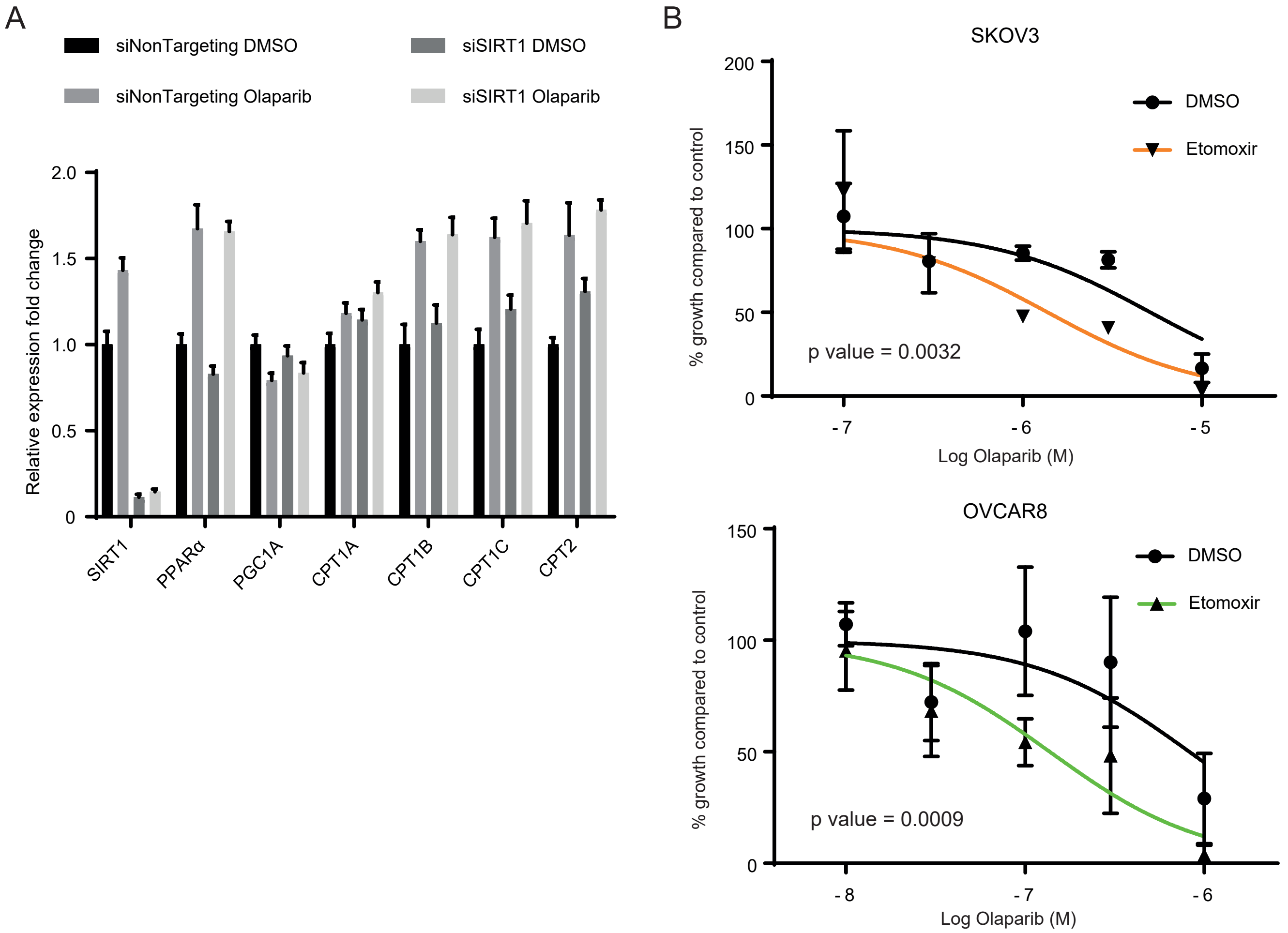
A
siNonTargeting DMSO
siSIRT1 DMSO
siNonTargeting Olaparib
siSIRT1 Olaparib
Relative expression fold change
2.0
1.5
1.0
0.5
0
SIRT1
PPARα
PGC1A
CPT1A
CPT1B
CPT1C
CPT2
B
SKOV3
200
150
100
50
0
% growth compared to control
DMSO
Etomoxir
p value = 0.0032
- 7
- 6
- 5
Log Olaparib (M)
OVCAR8
150
100
50
0
% growth compared to control
DMSO
Etomoxir
p value = 0.0009
- 8
- 7
- 6
Log Olaparib (M)

Table list

Table S1. Patient information of OXO-PCR subjects (see Excel file).

Table S2. Pseuodotime analysis and DE genes (see Excel file).

Table S3. DE genes between “Exceptional Responders” pre-chemo and "Exceptional Responders" post-chemo (see Excel file).

Table S4. DE genes between “Exceptional Responders” post-chemo and "Poor Responders" post-chemo (see Excel file).

Supplementary video

A video demonstrating complete clinical response in a patient (1016) who was one of the exceptional responders. Note the surgical clip in the post-chemotherapy video. The clip was used to mark the site from which a biopsy was obtained prior to chemotherapy and to guide selecting the post-chemotherapy biopsy site.
